# Viral and Prion Infections Associated with Central Nervous System Syndromes in Brazil

**DOI:** 10.3390/v13071370

**Published:** 2021-07-15

**Authors:** Ivanildo P. Sousa, Flavia B. dos Santos, Vanessa S. de Paula, Tuane C.R.G. Vieira, Helver G. Dias, Caroline A. Barros, Edson E. da Silva

**Affiliations:** 1 Enterovirus Laboratory, Oswaldo Cruz Institute, Oswaldo Cruz Foundation, Rio de Janeiro 21040-900, Brazil; edson@ioc.fiocruz.br; 2 Viral Immunology Laboratory, Oswaldo Cruz Institute, Oswaldo Cruz Foundation, Rio de Janeiro 21040-900, Brazil; flaviab@ioc.fiocruz.br (F.B.d.S.); helvergd@gmail.com (H.G.D.); 3 Molecular Virology Laboratory, Oswaldo Cruz Institute, Oswaldo Cruz Foundation, Rio de Janeiro 21040-900, Brazil; vdepaula@ioc.fiocruz.br; 4 Institute of Medical Biochemistry Leopoldo de Meis, National Institute of Science and Technology for Structural Biology and Bioimaging, Federal University of Rio de Janeiro, Structural Biology Program, Rio de Janeiro 21941-902, Brazil; tuane@bioqmed.ufrj.br (T.C.R.G.V.); carol.augustobarros@gmail.com (C.A.B.); 5 Federal Institute of Rio de Janeiro-PMBqBM, Rio de Janeiro 20270-021, Brazil

**Keywords:** enteroviruses, flaviviruses, alphaviruses, herpesviruses, prion, central nervous system

## Abstract

Virus-induced infections of the central nervous system (CNS) are among the most serious problems in public health and can be associated with high rates of morbidity and mortality, mainly in low- and middle-income countries, where these manifestations have been neglected. Typically, herpes simplex virus 1 and 2, varicella-zoster, and enterovirus are responsible for a high number of cases in immunocompetent hosts, whereas other herpesviruses (for example, cytomegalovirus) are the most common in immunocompromised individuals. Arboviruses have also been associated with outbreaks with a high burden of neurological disorders, such as the Zika virus epidemic in Brazil. There is a current lack of understanding in Brazil about the most common viruses involved in CNS infections. In this review, we briefly summarize the most recent studies and findings associated with the CNS, in addition to epidemiological data that provide extensive information on the circulation and diversity of the most common neuro-invasive viruses in Brazil. We also highlight important aspects of the prion-associated diseases. This review provides readers with better knowledge of virus-associated CNS infections. A deeper understanding of these infections will support the improvement of the current surveillance strategies to allow the timely monitoring of the emergence/re-emergence of neurotropic viruses.

## 1. Introduction

Globally, central nervous system (CNS) infections present a high rate of morbidity and mortality, mainly in children and the elderly, resulting in serious public health problems and having a major impact in developing countries [1,2]. Indeed, a recent report demonstrated that the incidence of CNS infections in low- and middle-income countries is 726 and 299 cases per 100,000 population, respectively, whereas the rate for high-income countries is 11 cases/100,000 population [2]. In addition to this difference in the CNS infection rate, other factors, such as a limited workforce, poverty, overcrowding in the public health system, and insufficient sanitation, can influence the persistence and spread of the infections [2]. A recent predictive model identified “hotspots” for emerging infectious diseases with the potential to induce CNS infections, such as viruses and prions [3]. Additionally, this study suggested that these hotspots are localized in developing countries, especially in tropical Africa, Latin America, and Asia [3]. As mentioned above, the incidence of CNS infections differs not only by geography, but also age groups and due to the capability of vectors to transit efficiently, as observed for arthropod-borne viruses.

**Figure 1:**
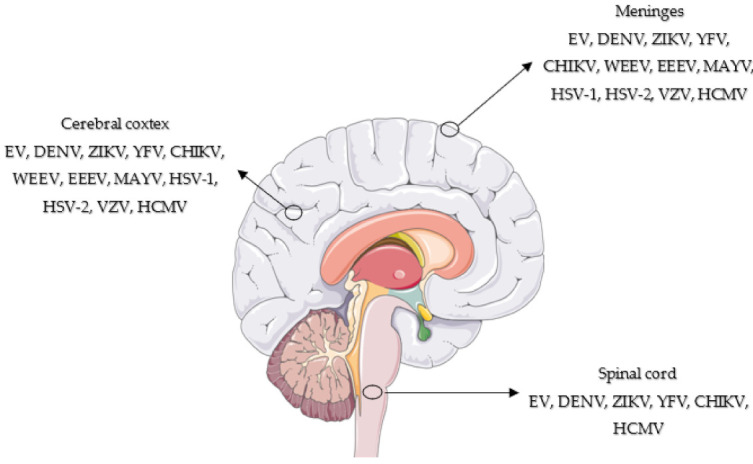
Brain areas commonly associated with viral infection. Enterovirus (EV); Dengue virus (DENV); Chikungunya virus (CHIKV); Human cytomegalovirus (HCMV); Eastern equine encephalitis virus (EEEV); Herpes simplex virus-1 (HSV-1); Herpes simplex virus-2 (HSV-2); Mayaro virus (MAYV); Varicella-zoster virus (VZV); Western equine encephalitis virus (WEEV); Yellow fever virus (YFV); Zika virus (ZIKV).

Many CNS infections are caused by bacteria, fungi, parasites, prions, and viruses and are an ongoing concern due to increased migration, tourism travel, and the ease with which people are displaced. The ongoing pandemic caused by the SARS-CoV-2 virus is a devastating example [4]. Regarding viral infections, a broad range of viruses has been reported as the major causal agents in many CNS infections, causing diseases ranging from febrile illness to infection that can affect distinct anatomical regions, such as the meninges (meningitis), brain (encephalitis), and spinal cord (myelitis) [5], or simultaneously in multiple regions (meningoencephalitis, encephalomyelitis) (Figure 1).

Additionally, the vast majority of viral infections present at least one neurological symptom during the acute phase, which can lead to neurological, behavioral, and cognitive sequelae months after the initial infection [2,6]. A recent report demonstrated that children (0.5–8 years) with severe viral CNS infections show enhanced risk of psychotic illness when they reach young adulthood [7]. Furthermore, the recent upsurge caused by the Zika virus revealed that the viral infection during the first part of fetal life could lead to severe brain malformations [6]. CNS viral infection may also be linked to other disorders that are rarely related, such as memory deficits, amyotrophic lateral sclerosis, and Alzheimer’s disease [6,8,9,10].

There is increasing interest in the characteristics and mechanisms associated with viral CNS infections to understand how a wide variety of viruses were selected throughout evolution to gain access to the CNS and to induce effective infection at different stages of life. The different replication strategies adopted for each virus and the poor nucleic acid polymerase fidelity observed in RNA viruses could favor the rise of cumulative mutations and/or recombinations and the emergence or re-emergence of neurotropic viruses of unpredictable pathogenicity and clinically unknown outcomes. Interestingly, several studies have demonstrated that the role of commensal microbiota and the CNS (the so-called gut-brain axis) is associated with viral infection [11,12,13]. However, CNS infections are not consequences of a single process, and studies are required to better understand the mechanisms and main factors associated with viral neurotropism. 

Overall, the incidence of CNS infections is underestimated due to limitations in diagnostic tools and poor demographic information, particularly in low- and middle-income countries [2]. Although Brazil has accumulated information related to viral infections of the CNS, little is known about the geographical distribution and molecular and epidemiological aspects of the viruses circulating in the country. Therefore, a better understanding on the patterns of viral dissemination within and between countries and continents is critical to devising global surveillance initiatives. The current review addresses this question, providing an overview of epidemiological patterns and genetic diversity of the most common viral infections associated with CNS in Brazil. These include enteroviruses, arboviruses (*Flaviviridae* and *Togaviridae* families), and viruses from the *Herpesviridae* family (Table 1). Because viruses and prions are closely related, we also highlight important aspects of prion-associated diseases, which are rarely reported and lacking information in Brazil. The presence of prion domains (domains enriched in asparagine and glutamine) has been reported in different viral families [14].

## 2. Viruses Commonly Associated with CNS Infection in Brazil

### 2.1. Enteroviruses and Enterovirus Transmission

Enteroviruses (EVs) are nonenveloped, small, icosahedral-shaped viruses which have a capsid composed of four structural proteins (VP1–VP4), with single-stranded positive-sense RNA, belonging to the *Picornaviridae* family. Based on their molecular and biological properties, they have been classified into 15 species: EV A–L and human rhinoviruses A–C. However, only EV A to D species and rhinoviruses are known to cause human infection. These viruses are transmitted primarily by the fecal–oral route and are able to advance from the primary site of infection (gastrointestinal tract) to other tissues. Some EV species (e.g., rhinoviruses and EV-D68) can also exploit the respiratory route and spread through the respiratory secretion. Additionally, EVs that cause a vesicular exanthem, as observed in hand, foot, and mouth disease (HFMD), can spread through direct or indirect contact with contaminated discharges or the fluid of blisters from infected people, or objects that contain an infectious virus. Exceptions are the EVs associated with acute hemorrhagic conjunctivitis (EV-D70 and CVA24) that can also be transmitted by direct or indirect eye–hand–fomite contact. Enteroviruses are distributed globally, particularly in tropical regions where poor hygiene conditions play a critical role in the efficiency of viral transmission.

#### 2.1.1. Epidemiology of the EV Associated with CNS

EVs enter host cells by binding to one or multiple receptors (e.g., ICAM-1, CAR, DAF, and CD155), followed by receptor-mediated endocytosis and RNA release [15]. EVs are responsible for a broad-spectrum of diseases, such as the common cold; hand, foot, and mouth disease; myocarditis; and upper and lower respiratory tract infection. Although EV infections are mostly asymptomatic, these viruses can cause severe CNS disease, particularly aseptic meningitis (AM), acute flaccid paralysis (AFP), encephalitis, and acute flaccid myelitis (AFM) [16]. To gain access to the CNS, EVs must cross the blood–brain barrier (BBB), infecting brain endothelial cells or immune cells, using the latter as vehicles in a mechanism known as the “Trojan Horse” [15,17,18]. Additionally, EVs can spread via neuromuscular junctions from muscles to motor neurons through retrograde axonal transport [15,17]. A recent review by Majer and colleagues [18] discussed the pathways exploited by the EVs to counteract the BBB and gain access into the CNS. Moreover, the cellular mechanisms of enterovirus neuropathogenicity and immunology evoked by EVs during CNS infection, as well as the mechanisms of neuronal cell death, have also been reviewed.

Globally, EVs have been responsible for the majority of aseptic meningitis/encephalitis cases, particularly in children [19,20]. Poliovirus is the main neurotropic EV because it is associated with poliomyelitis cases in children. However, due to the advanced stage of polio eradication, the role of non-polio enteroviruses (NPEVs) associated with AFP cases, in addition to other CNS infections, have been highlighted [16,17,21]. In addition to poliovirus, EV types have been associated with several CNS infections, such as AFP, AFM, AM, and encephalitis. The most common are EV-A71, CVA2, and CVA4 (EV-A species); CVB3, CVB5, E6, E7, E11, E13, and E30 (EV-B species); CV-A24 (EV-C species); and EV-D68 (EV-D species) [16,21]. This wide diversity of EV types related to CNS infections highlights their high tropism to neuronal cells and neuropathogenic potential.

#### 2.1.2. Enteroviruses Associated with CNS in Brazil

Brazil has accumulated significant information regarding WPV and vaccine-derived polioviruses (VDPV). In addition, the molecular diversity and epidemiological features of other EV types related to CNS infections circulating in the country have also been highlighted in recent years [22,23,24,25,26]. As previously reported in other countries, aseptic meningitis is the principal neurological disease associated with NPEV in Brazil [27,28]. Globally, E30 and E6, followed by E13, are the most prevalent EV types identified to cause epidemics/sporadic cases [29]. In Brazil, numerous studies have demonstrated that E30 is the main EV type associated with AM cases in different geographic areas of the country, followed by E6 [23,30,31,32]. Although E13 has been found to be responsible for an AM outbreak, its detection in Brazil is not common [23,33] compared to the observations in other regions such as Europe, America, and the Western Pacific [29]. Furthermore, although other EV types have been identified in recent years in Brazil, four important EV types belonging to EV-B species should be highlighted—CVB5, E7, E11, and E18—because these viruses are also associated with a high detection rate in AM cases in Brazil [23,25,30]. It is also notable that EV-A and EV-C species are rarely associated with AM cases globally.

Another remarkable observation concerns EV types involved with AFP cases in Brazil. Although polioviruses are the main causative agent of AFP, a significant proportion of NPEVs has been associated with this neurological disorder [21]. The last case of paralytic poliomyelitis caused by wild poliovirus in Brazil occurred in 1989. However, the identification of a rare recombinant type3/type2 Sabin-related poliovirus isolated from an AFP case has recently been reported as an adverse vaccine event [22]. Surveillance data shows that in Europe, the USA, and Africa, most cases have been associated with EV-B species, whereas EV-A species are normally more prevalent in Asia, where they manifest primarily as hand, foot, and mouth disease [21,34,35]. In Brazil, EV-B species are the most frequently detected in AFP cases, followed by EV-A and EV-C species [23]. CVA2 and EV-A71 (EV-A species); CVB5, E6, E7, and E11 (EV-B species); and CVA13 and EV-C99 (EV-C species) are the most commonly isolated EV types [24]. According to previous studies, EV-A71 has been the most commonly detected NPEV associated with AFP cases, followed by E6 and E11 [21]. This detection pattern was also similarly reported in AFP patients in Brazil [24]. In contrast, CVA2 is an EV type with a low involvement in AFP syndrome but has shown a high detection rate in Brazil [21,24]. This possibly enhanced neurotropism could be due to an amino acid substitution inside the BC loop, an important immunogenic site localized in VP1 proteins [24]. Further studies must be conducted to understand this different pattern of detection.

Although the major causative agents of encephalitis/meningoencephalitis are herpesviruses and arboviruses, particularly in developing countries, EVs have also been found to be among the most frequent pathogens associated with encephalitis globally [16,17,36,37]. The vast majority of the existing encephalitis/meningoencephalitis cases have been associated with EV-A71, followed by E30 and E18 [29]. In the specific case of Brazil, EVs have played a major role in these CNS infections [25,38], and the most frequent encephalitis-associated enteroviruses found have been CVB2, E6, E18, and E30 [25,39]. 

Overall, these scenarios suggest different circulation patterns of viruses associated with different neurological diseases in Brazil compared to other countries. Furthermore, it is worth noting that the cocirculation of a high number of different EV types can lead to recombination/mutation events, favoring the emergence/reemergence of novel EV types with unknown pathogenicity. EV-A71 has been identified as a major EV type associated with AFP, although it represents less than 1% of all EV types associated with aseptic meningitis in Brazil [22], and E6 has played a relevant role in neurological disorders, particularly AFP and aseptic meningitis cases [23,24]. The above information highlights the need to conduct further studies to better understand the circulation and the mechanisms of EVs, in addition to the main factors associated with enteroviral neurotropism.

#### 2.1.3. Diagnosis of Enteroviruses

As recommended by World Health Organization (WHO) in its guidelines for enterovirus surveillance, as support for the Polio Eradication Initiative, specimens of feces and cerebrospinal fluid (CSF) and nasopharyngeal and oropharyngeal swabs should be collected from patients for EV detection [40]. Fecal samples are the preferred specimens regardless of clinical presentation. Reverse transcriptase PCR (Rt-PCR) targeting the highly conserved 5’non-coding region (5’NCR) can be used for diagnosis of EVs because it offers several potential advantages for detection and diagnosis of EVs such as sensitivity, specificity, and short turnaround time. For AFP surveillance, the WHO has established a standard procedure for EV isolation through inoculation of stool specimens on susceptible cultured cells.

### 2.2. Arboviruses

Arthropod-borne viruses (arboviruses), a taxonomically diverse group of viruses transmitted between arthropod vectors and vertebrate hosts, are a significant public health problem globally. The most studied arboviruses include the viruses belonging to the families *Flaviviridae* (Dengue virus (DENV), Zika virus (ZIKV), Yellow Fever virus (YFV), Saint Louis Encephalitis virus (SLEV), West Nile virus (WNV), Japanese Encephalitis virus (JEV), Tick-Borne Encephalitis virus (TBEV), Rocio virus (ROCV), Cacipacore virus (CPCV), Ilheus virus (ILHV), Bussuquara virus (BUSV), and Iguape virus (IGUV), *Togaviridae* (Mayaro virus (MAYV), Chikungunya virus (CHIKV), and Eastern Equine Encephalitis virus (EEEV)), and Peribunyaviridae (Oropouche virus (OROV)). Five human arboviruses have emerged or re-emerged in both hemispheres in recent decades: The flaviviruses DENV, ZIKV, WNV, and YFV, and the alphavirus, CHIKV [41,42]. Moreover, some of the flaviviruses pathogenic to humans are endemic in many parts of the world, including Brazil.

Commonly observed clinical manifestations of arboviruses infections in humans range from undifferentiated, moderate, or severe febrile disease; rashes; and arthralgia to neurological and hemorrhagic syndromes. The febrile illness is usually characterized by fever, headache, retro-orbital pain, and myalgia, whereas the neurological syndromes can include meningitis, myelitis, encephalitis, paralysis, paresis, seizures, problems associated with coordination, and behavioral changes [43].

According to the Brazilian Ministry of Health, the neuro-invasive arboviruses of major public health importance in Brazil are those belonging to the genera *Flavivirus*, *Alphavirus*, and *Orthobunyavirus*, particularly DENV, CHIKV, and ZIKV [44]. Since 2015, DENV, ZIKV, YFV, and CHIKV have cocirculated in Brazil, representing a challenge not only for diagnosis, but also for vector control and clinical and therapeutic management. Here, these three flaviviruses and their association with CNS disease are briefly addressed.

#### 2.2.1. Flaviviruses

The flaviviruses addressed in this review are mainly transmitted to humans through the bites of infected *Aedes* mosquitoes in two distinct transmission cycles: A sylvatic cycle maintained between mosquitoes and nonhuman primates, and an urban cycle between humans and domestic *Aedes* spp., such as *Aedes aegypti*, the main vector [45]. Flaviviruses are small (with a diameter of approximately 50 nm), enveloped, positive-stranded RNA viruses, with the viral genome encoding three structural proteins—C (capsid), E (envelope), and M (membrane)—and seven nonstructural proteins (NS1, NS2A, NS2B, NS3, NS4A, NS4B, and NS5) [46,47].

The flaviviruses’ evolution and epidemiology are mainly determined by the ecological needs of their invertebrate and vertebrate hosts, and they can be grouped by their genome homology, antigenicity, pathogenicity, geographic distribution, and ecological associations [48].

Most flaviviruses infections in humans (~80%) are asymptomatic or result in mild manifestations, such as fever, arthralgia, myalgia, headache, retro-orbital pain, exanthema, and conjunctivitis. However, in some cases, they may cause systemic, hemorrhagic, and neurological syndromes once they reach the CNS. The most severe clinical features related to encephalitis and other neurological syndromes are mostly caused by JEV, WNV, TBEV, and ZIKV [49,50,51], whereas severe systemic and vascular manifestations are mainly related to DENV and YFV infections [52,53,54,55,56].

In Brazil, DENV, ZIKV, and YFV are the most important flaviviruses and have caused explosive and severe epidemics in recent years. Therefore, we chose to address these viruses in this review. Moreover, in endemic countries for arboviruses, such as Brazil, where individuals are highly exposed, these viruses may be involved in CNS-associated disease, although these are considered to be rare events [43,44].

Despite previous efforts, knowledge regarding the pathogenic mechanisms involved in the neurological syndromes caused by flaviviruses is still scarce. It is known that flaviviruses can cause neurological disease by directly attacking the nervous system or indirectly via immune-mediated processes [57]. As previously reported in several studies and as reviewed by the authors of [56], the flaviviruses JEV, WNV, ZIKV, YFV, DENV, and TBEV are believed to reach the CNS by crossing the blood–brain barrier (BBB) [58,59,60]. However, the role of the BBB breakdown for virus entrance into the brain is still not fully understood. Although flaviviruses are not always neuro-invasive, they are able to infect neurons [61,62]. It has been shown that flavivirus neuropathogenesis is related to the apoptosis of infected neuronal cells and/or the immune response induced by the immune cells, because factors produced by microglia may be toxic to neurons [63,64,65]. 

A recent review by Mustafa and colleagues [56] discussed the pathways exploited by the flaviviruses to counteract the BBB and infect the CNS. Viral replication in the brain endothelial cells, leading to the downregulation of tight junction proteins and thus increasing barrier permeability, has been shown in vitro. It has been also reported that astrocytes, microglia, and other BBB-associated cells, upon viral replication and activation, also play a role in the endothelial barrier permeability, because they are associated with the expression and secretion of inflammatory mediators. Leukocyte infiltrates recruited to the CNS result in viral invasion via a Trojan Horse mechanism, contributing to the BBB breakdown and neurological alterations [56].

##### Dengue

Dengue is the most important mosquito-borne viral disease in humans. Dengue is caused by infection of any of four DENV serotypes (DENV-1 to DENV-4) and is associated with significant morbidity, mortality, and economic impact, particularly in developing countries. Each DENV serotype includes several genotypes, a group of DENV isolates that have no more than 6% nucleotide sequence divergence [66].

Dengue incidence has increased 30-fold in the last 50 years, and over 50% of the global population lives in areas at risk of infection by one of the four DENV serotypes. It has been suggested that DENV originated in nonhuman primates in Africa and Asia in a sylvatic cycle about 1000 years ago [66,67]. 

DENV infections may result in a wide spectrum of clinical manifestations, from a mild to a potentially life-threatening disease. According to the WHO (2009) criteria, the disease is currently classified as Dengue, Dengue with warning signs, and severe Dengue. Symptoms usually include fever, nausea, vomiting, rash, and aches. However, if untreated, the mortality rate of the severe disease can be as high as 20% [42,68].

Dengue is not considered a neurotropic virus. However, neurotropism and neuro-invasion have been reported previously [55,69], and encephalitis and encephalopathy are the most common neurological presentations of DENV infections [70,71,72]. It is estimated that 0.5–21% of DENV-infected patients develop neurologic manifestations, with DENV-2 and DENV-3 most frequently associated with cases of myelitis, meningitis, and encephalitis [70]. The clinical features, treatment, and possible pathogenesis of the main neurological complications of DENV infection and the peripheral nervous system have been reviewed by Li and colleagues [55].

Encephalitis with acute signs of cerebral involvement has been described in many studies [55,69,73,74,75,76]. Encephalopathy characterized by cognitive disorders, convulsions, mood, personality, and behavior disorders has also been extensively reported [55,69,74,77,78,79,80]. 

Dengue-related meningitis is rare, but it has been previously described in a few reports, including in children, for whom the acute onset of fever and symptoms such as headache, vomiting, and/or nuchal rigidity were described [28,65,73,81,82,83].

Ischemic and hemorrhagic strokes in conjunction with hemiparesis, dysarthria, headache, vertigo, vomiting, and somnolence have been described in a few cases [84,85]. A cases study by Vargas-Sánchez et al. [86] reported DENV-associated intracerebral hemorrhage in the pontine, basal ganglia, cerebellar, parietal, temporal, and frontal lobes of the brain. Dengue-related transverse myelitis, longitudinally extensive transverse myelitis, and acute disseminated encephalomyelitis have also been reported in some studies [69,87,88,89,90,91].

Peripheral nervous system (PNS) involvement in DENV infection occurs in 5% of neurological symptoms, and includes Guillain-Barré syndrome (GBS), which is not commonly observed; hypokalemic quadriparesis or plegia; mononeuritis multiplex; brachial plexitis; diaphragmatic paralysis; and myositis [80,92,93,94,95,96]. GBS is an immune-mediated disorder of the PNS that manifests as acute onset of ascending paralysis and sensory symptoms [97], and is the most common cause of acute flaccid paralysis globally [98].

##### Zika

Although first isolated in 1947 [99], ZIKV became an emerging public health problem after the outbreak in 2015 in Brazil, during which viral infections were associated with severe neurological complications, such as microcephaly in the fetus and GBS in adults, leading the WHO to declare a public health emergency of international concern in 2016 [100]. Phylogenetic analysis of ZIKV showed that the virus is spread in three lineages: West African, East African, and Asian [101].

As an arbovirus, ZIKV is mainly transmitted by the bites of infected *A. aegypti* mosquitoes. However, vertical transmission, from mother to fetus, can also occur. Vertical transmission been associated not only with spontaneous abortions and stillbirth but also with congenital malformations in the fetuses and/or newborns, for which symptoms are not limited to microcephaly. Congenital Zika virus syndrome (CZS) is mainly characterized by signs and symptoms such as microcephaly, parenchymal or cerebellar calcifications, ventriculomegaly, CNS hypoplasia or atrophy, arthrogryposis, focal pigmentary retina mottling, chorioretinal atrophy and/or coloboma, pallor, atrophy, increased excavation, hypoplasia and/or coloboma of the optic nerve and abnormal visual function, and low birthweight for gestational age, as reviewed in Freitas and colleagues [102].

It is estimated that 80% of ZIKV infections are asymptomatic. When symptomatic, the disease is similar to but usually milder than Dengue, and is characterized by fever, rash, arthralgia, and conjunctival hyperemia [103]. The clinical manifestations are usually mild and self-limiting [104,105]. However, severe neurological manifestations and an increased number of GBS cases were reported in the 2013 French Polynesia outbreak [106,107]. In fact, following the spread of ZIKV, countries and territories, including Brazil, have reported an increased incidence of GBS cases following recent ZIKV infection [108,109,110,111]. It has been shown that GBS is slightly more common in men than in women, and limb weakness and areflexia/hyporeflexia are the most frequent clinical findings [112].

In a single-center Brazilian study, ZIKV infection was associated with an increased incidence of a wide spectrum of serious neurologic syndromes, some of which lead to death, such as GBS, encephalitis, transverse myelitis, and chronic inflammatory demyelinating polyneuropathy [113].

The mature adult brain appears to be less susceptible to the possible neuro-invasive effect of ZIKV. Although CZS has been associated with encephalitis and microcephaly in fetuses and newborns, a few cases of CNS involvement, such as encephalopathy, encephalitis, meningitis, myelitis, and/or seizures, have been reported in adults. The ZIKV- related CNS complications appear to have a predominantly neuroinflammatory profile [112,114,115,116,117].

##### Yellow Fever

YFV is the etiological agent of yellow fever, an acute hemorrhagic disease with a significant impact on public health that is endemic in tropical regions in Africa and South America. Approximately 200,000 YFV infections and 30,000 deaths occur annually, mainly (90%) in Africa. Despite the availability of a safe and efficient vaccine for more than 85 years, an increased number of YF outbreaks have been reported recently in Africa and Brazil [118].

In Brazil, since the last urban cycle transmission was reported in 1942, YFV infections have occurred due to the sylvatic cycle. YFV infections have mainly been confined to the Amazon region, where transmission is maintained between sylvatic mosquitoes (*Haemogogus* and *Sabethes genera*) and nonhuman primates and where humans are incidental hosts [119,120]. Outbreaks re-emerge from the forest every 7–8 years, and, during the 2016–2019 outbreak in Brazil [121], the virus spilled outside the Amazon basin to areas of naïve populations [122,123,124,125]. YFV has one serotype but is divided into four genotypes—West and East African, South America I, and South America II—and several lineages [126,127].

Factors such as inadequate vaccination coverage, inability to control sylvatic transmission cycles, reinfestation of major urban centers by *A. aegypti*, and inadequate vector control approaches influence the potential re-emergence of YFV in urban areas [124].

Most YFV infections are asymptomatic (nearly 90%) or present as a mild undifferentiated febrile illness characterized by an abrupt onset of fever, headache, photophobia, lumbosacral pain, nausea, prostration, myalgias, facial flushing, and conjunctivitis. In severe cases, clinical manifestations such as jaundice, albuminuria, oliguria, bradycardia, delirium, stupor, and metabolic acidosis, leading to shock and hemorrhage, may occur. About 15% of cases do not recover and progress to an intoxication phase with high fever, multisystem organ failure, and fatality [128].

Although rare, YFV infection may induce encephalitis. However, neurological complications after wild-type YFV infection, signs of CNS involvement, and generalized seizures have been reported in the past [129]. 

Adverse vaccine events have been reported and associated with viscerotropic disease, which presents signs and symptoms that are not distinguished from the natural infection, and neurological disease [130,131,132,133,134,135].

A study on neurological adverse events following administration of the 17DD yellow fever vaccine in Brazil found the highest rate of neurological adverse events in children aged 5 to 9 years old, with a rate of 0.83 per 100,000 doses in national analysis [136]. Among the adverse neurological events, GBS, acute disseminated encephalomyelitis cases, transverse myelitis, bilateral optic neuritis, and meningoencephalitis with polyradiculoneuritis and Kinsbourne syndrome have been reported [137]. There is no specific treatment for adverse events associated with the yellow fever vaccine [138]. Because the lethality of YFV infections is significantly higher than the incidence of side effects that may be triggered by the vaccination, vaccination against yellow fever is strongly recommended in areas at risk [139].

Overall, the diagnosis of CNS viral infection involves a combination of clinical findings, molecular and serological assays in CSF, neuroimaging, and chemocytological analysis [140], as reviewed by the authors of [65]. According to the criteria of the Brazilian Ministry of Health and the Centers for Disease Control and Prevention for defining cases of neuro-invasive arbovirus, confirmed cases of neuro-invasive arbovirus are those with viral detection by RT-PCR in serum or CSF and/or the detection of IgM antibodies in CSF by enzyme immunoassay (ELISA), with the exclusion of endemic arbovirus [44,141], because neurologic symptoms are not specific to arboviruses. 

IgM ELISA is commonly used to diagnose flaviviruses in the CNS, because virus-specific IgM in CSF represents intrathecal synthesis as IgM is a pentamer and is too large to cross the blood–brain barrier [142]. Therefore, the detection of viral RNA and specific IgM in CSF are indicative of the viral infection in the CNS. The combined use of molecular and immunologic tests in CSF/serum might provide greater support for the diagnosis of neurologic disorders caused by arboviruses in endemic areas [72].

Viruses can reach the brain through different mechanisms, including peripheral nervous system and axonal transport and the hematogenous route. As described here and based on several reports, it has been shown that, in general, flaviviruses can be neurotropic and neurovirulent. However, the pathways by which these viruses reach and invade the CNS are yet to be fully understood. Significant research has been conducted so far, but more investigations are needed to elucidate the neuropathogenic mechanisms and the resulting outcomes.

#### 2.2.2. Alphaviruses

The genus *Alphavirus* belongs to the *Togaviridae* family, which currently comprises 31 recognized species divided into 8 complexes based on antigenic relationships. Alphaviruses are enveloped with icosahedral capsid and single-stranded positive-sense RNA genomes of about 12 kilobases with 2 open reading frames (ORFs), which encode the nonstructural (ns) or replicase polyprotein and the structural polyprotein [143].

CHIKV, Mayaro virus (MAYV), Ross River virus (RRV), and Sindbis virus (SINV), which are collectively known as Old World alphaviruses, tend to cause a clinical syndrome characterized by fever, rash, and arthritis, whereas the New World alphaviruses—Venezuelan equine encephalitis virus (VEEV), eastern equine encephalitis virus (EEEV), and western equine encephalitis virus (WEEV) [144]—are generally associated with encephalomyelitis [144]. Among the alphaviruses, CHIKV is the most important in Brazil, causing explosive epidemics since its introduction in late 2014.

##### Chikungunya

CHIKV was initially identified in 1952 in Tanzania but spread throughout the Indian Ocean region in the mid-2000s and was introduced into the Caribbean in 2013 [145]. There is only one serotype, and four distinct genotypes have been described: West African, East-Central-South-African (ECSA), Asian, and Indian Ocean Lineage (IOL). The IOL emerged as an ECSA monophyletic group [146].

The local transmission of CHIKV in Brazil was first detected in 2014 in Oiapoque, Amapá [146]. Since then, cities in all regions of the country have reported epidemics, and both Asian and ECSA genotypes have circulated in the country [147]. With the exception of the Chikungunya virus (CHIKV), alphaviruses associated with neurological disorders in Brazil are transmitted by sylvatic mosquitoes in enzootic cycles, where man is an accidental host [148]. CHIKV is transmitted in urban areas by the anthropophilic vectors *Aedes aegypti* and *A. albopictus* [149].

CHIKV infection typically induces a mild disease in humans, characterized by fever, arthralgia, myalgia, and rash. Moreover, long-term sequelae can occur after infection. During the CHIKV epidemic in Brazil, atypical and severe cases were reported, including neuronal impairment in adults and newborns (mother-to-child infection) [149,150,151,152,153]. 

CHIKV-associated neurological disorders were first described in the 1960s in Asian countries [154,155]. The origin and pathophysiology of neurological manifestations from CHIKV infection remain poorly understood. Studies developed in animal models have suggested that the virus specifically targets the choroid plexuses and the leptomeninges in the CNS [156]. Additionally, immunohistochemistry of the infected mouse brain demonstrates that the antigen is detected in the cortex and thalamus [157]. In a neuro-invasion zebrafish model, macrophages played no role in the entry of CHIKV into the CNS. However, CHIKV infects the brain microvascular endothelium of the BBB, suggesting that this can be the entry route to the CNS [158].

It has been shown that neurological disease associated with CHIKV infections has the highest mortality rate of potential atypical disease manifestations [159,160], and neurological complications following CHIKV infection are characterized by elevated TNF-α, IFN-α, IL-6, IL-8, CCL2, CCL5, CCL17, and CXCL9 in CSF in comparison to patients without neurological disease [161].

Several reports have described the occurrence of neurological complications associated with CHIKV infection in Brazil [57]. The frequency of GBS is high in adults, and higher than that observed in neurological cases associated with Dengue or Zika in the same period [72,162]. From a retrospective investigation carried out in fatal Chikungunya cases in northeastern Brazil, it was identified that, of the 39 patients with neurological complications, CHIKV-RNA was detected in 92.3% of the CSF samples [153].

Neuro-invasive CHIKV infections do not have specific symptoms, which can cause difficulty in clinical diagnosis [151]. The neurological disease and syndromes associated with CHIKV most reported in Brazil are encephalopathy, myelopathy, encephalomyelopathy, neuropathy, and myeloneuropathy. Symptomatology may include confusion, seizures, drowsiness, areflexia, paresthesia, urinary retention, dysarthria, headache, neck stiffness, dyspnea, and spastic paraparesis. More serious conditions may require ventilation and other intensive care [163,164,165,166]. Although it has been scarcely studied, Chikungunya is also related to the onset of psychiatric symptoms [167].

The diagnosis of CHIKV is based on molecular (RT-PCR) or serological (e.g., ELISA IgM/IgG) methods from blood or CSF samples. Neurological impairment can be detected with the support of CSF laboratory analyses (pleocytosis is a strong indication) in addition to brain imaging and an electroencephalogram [72,168,169].

Management for neurological complications depends on the symptoms presented. However, antivirals have been used, mainly acyclovir, in addition to intravenous immunoglobulin, plasmapheresis, and corticoids [151,168,170,171]. 

##### Other Alphaviruses

Other alphaviruses, such as Western equine encephalitis (WEEV) and Madariaga virus, also known as South American eastern equine encephalitis virus, are enzootic pathogens in Brazil with significant potential for neurological disease in both humans and horses. However, outbreaks of equine encephalitis in Brazil are mostly epizootic, with isolated human cases in recent decades. A single case of encephalitis by EEEV was reported in Bahia, Northeast Brazil, in the 1950s [172]. No human case of WEEV has been diagnosed in Brazil to date, but serological evidence is frequently reported in wild and domestic animals [173]. MAYV is an important epidemic agent in the north and central-west regions of Brazil, but cases associated with neurological disease are rare or absent [174].

The implementation of arbovirus neuro-invasive disease surveillance programs has contributed to improving the notification and investigation of cases, but most laboratories still do not routinely diagnose those alphaviruses.

### 2.3. Alphaherpesviruses

Herpes simplex virus (HSV)-1, herpes simplex virus (HSV)- 2 (HSV-2), and varicella-zoster virus (VZV) are members of the alphaherpesvirus subfamily of viruses commonly identified as causing encephalitis [175]. All of them contain a double-strand DNA genome: HSV-1 with 152 kb, HSV-2 with 155 kb, and VZV with 125 kb. The alphaherpesviruses are characterized by a short replication cycle, rapid destruction of the host cell, and the ability to establish latency. 

Infection with herpes simplex virus, commonly known as herpes, can be due to either herpes simplex virus type 1 (HSV-1) or herpes simplex virus type 2 (HSV-2). HSV-1 is mainly transmitted by oral-to-oral contact, causing infection in or around the mouth (oral herpes). However, HSV-1 can also be transmitted through oral–genital contact to cause infection in or around the genital area (genital herpes). HSV-2 is almost exclusively transmitted through genital-to-genital contact during sex, causing infection in the genital or anal area (genital herpes). Although HSV encephalitis (HSE) is a rare disease with an estimated global incidence of 1 case per 250,000–500,000, it is the most common cause of encephalitis among the *Herpesviridae* family. HSV-1 and HSV-2 are important causes of CNS infection in the setting of neonatal herpes [176,177]. Approximately one-third of HSE cases are considered a consequence of the immediate CNS invasion through the trigeminal nerve or the olfactory tract after an episode of primary HSV-1 infection of the oropharynx [178]. However, the access route of HSV to the CNS remains controversial, and it is believed that primary and recurrent infections by HSV can cause encephalitis. Acute inflammation and hemorrhage are seen in the temporal lobes, and usually occur asymmetrically in adults and more diffusely in newborns [179].

It is believed that the reactivation of HSV in the peripheral ganglia with axonal transport to the temporal lobe and the reactivation of the virus in the brain is a possible cause of invasion in the CNS after an episode of recurrent HSV infection with subsequent spread [180]. About 30% of HSE cases are related to primary HSV infection (most commonly in children and adolescents), whereas 70% of cases are attributed to HSV reactivation. The clinical characteristics do not distinguish between HSE caused by primary infection or reactivation. During acute HSE, exuberant immune responses can contribute to CNS pathology [176,177,179].

The varicella-zoster virus (VZV) is also responsible for a wide range of CNS manifestations. VZV can cause a wide variety of symptoms. After the primary infection (chickenpox), the virus lies dormant in the nerves, including the cranial nerve ganglia, dorsal root ganglia, and autonomic ganglia. Many years after the person has recovered from chickenpox, VZV can reactivate to cause neurological conditions [181]. VZV reactivation can also cause disease of the central CNS, such as encephalitis. In addition, a VZV infection of the cerebral arteries produces vasculopathy, which can manifest itself as an ischemic stroke. Vasculopathy and ischemic stroke can occur after primary infection or reactivation of VZV when cerebral artery infection occurs [181]. 

The Alphaherpesviruses have a high prevalence in developing countries, with prevalence higher than 60% in adults. Encephalitis caused by HSV is the most common cause of sporadic fatal encephalitis globally, and CNS involvement is observed in approximately one-third of neonatal HSV infections. Most patients with encephalitis associated with HSV-1 or HSV-2 in primary infection are younger than 18 years of age. The infection associated with VZV usually occurs in patients of older ages after reactivation episodes [176,177,179].

In Brazil, a limited number of studies have reported the detection of HSV-1, HSV-2, and VZV in infections associated with the CNS. A study conducted in the Western Brazilian Amazon that investigated CSF samples from 165 patients with suspected CNS viral infection through polymerase chain reaction (PCR) found herpes simplex virus (HSV-1) (4.1% of patients), HSV-2 (4.1%), and VZV (20.4%) [27]. In Ribeirão Preto, Brazil, 200 CSF samples were taken from patients with clinically suspected viral CNS infection, and rates of 5% for HSV-1 and 0.5% for VZV were reported [182].

Whole-genome sequencing has been successfully applied for genotyping of HSV-1 [183,184,185,186], HSV-2 [187], and VZV [188]. It was concluded that HSV-1 genotypes are strongly connected to geographic regions and could be used to track early human migration patterns [189]. Sequencing can be performed to diagnose drug-resistant infections caused by HSV or VZV isolates. Molecular biology-based systems for the generation of recombinant viruses have been developed to link unknown mutations with their drug phenotypes. Fast and sensitive methods based on next-generation sequencing may improve the detection of heterogeneous viral populations of drug-resistant viruses and their temporal changes during antiviral therapy, which may allow for better patient management. However, the interpretation of genotypic tests requires a database that links amino acid changes to mutations associated with natural polymorphisms or drug resistance. The monitoring of drug-resistant heterogeneous viral mutant populations and their temporal changes during antiviral therapy should be associated with better patient management [190]. 

In recent years, single-gene inborn errors of innate immunity have been shown to be associated with susceptibility to HSV encephalitis. Regarding the clinical manifestation, HSV-1 causes more than 90% of cases of HSE in adults, whereas HSV-2 infection generally causes aseptic meningitis. In addition, HSV-2 is a common cause of generalized acute encephalitis in neonates [191]. Although focal neurological findings are seen in the acute phase of HSE, the initial symptoms are generally considered to be nonspecific and can occur in both CNS viral and bacterial infections. In ~80% to 90% of cases, headache and fever are highlighted. Other common features include altered levels of consciousness, focal or generalized seizures, cranial nerve deficits, hemiparesis, dysphasia, aphasia, and ataxia [192,193,194]. 

Whereas diagnostic studies are pending, the best approach to improving long-term outcomes is to include HSE in the differential diagnosis of every patient presenting with fever and neurologic findings and to rapidly initiate empiric intravenous acyclovir therapy. Detection of HSV DNA in the cerebrospinal fluid by PCR is the gold standard for the diagnosis of HSV encephalitis and neonatal meningoencephalitis. Temporal lobe abnormalities detected by brain imaging are considered strong evidence of HSE. PCR has overall sensitivity and specificity of >95% for diagnosis of HSE compared to brain biopsy [195]. Magnetic resonance imaging (MRI) is the preferred imaging technique for patients with suspected HSE. For VZV diagnosis, positive PCR findings are also a gold standard. However, a negative PCR result does not exclude the diagnosis of VZV vasculopathy. VZV DNA can be detected only during the first 2 weeks of the disease. The CSF viral load predicts an uncertain outcome, and there is no consensus between quantitation of HSV DNA in CSF and severity of clinical disease and prognosis [196,197]. Serological testing has also been used in diagnosis of HSV in CNS [198].

Acyclovir (ACV) is currently the antiviral drug approved by the FDA for the treatment of HSE with a reduction in mortality [199,200]. ACV treatment should discontinued with caution in patients with a strong suspicion of HSE. Cerebrospinal fluid PCR results may be negative in the course of herpes simplex encephalitis. Therefore, repeat cerebrospinal fluid analysis should be considered. Ideally, more than one diagnosis should be made before treatment is interrupted [201]. The use of corticosteroids to suppress inflammatory responses in the CNS during HSE and to reduce immune-mediated pathology has not yet reached a consensus. Oral ACV therapy is not recommended for the treatment of HSE because this application does not reach adequate levels in CSF. However, valacyclovir has good bioavailability and is converted to LCA after absorption. During acute HSE, exuberant immune responses may contribute to the CNS pathology, suggesting that selective immunosuppressive therapy, coupled with potent antiviral drugs, may eventually play a role in the therapeutic management of HSV [178]. VZV CNS infection is treatable with intravenous acyclovir and corticosteroids in addition to anticoagulants if a hypercoagulable state exists. Valaciclovir can be used orally after acyclovir treatment [181]. Attempts have been made to use immunoglobulin therapy with limited effects. Progressive multifocal leukoencephalopathy has been treated with an emerging immune activation therapy in a limited number of patients with incomplete success [202].

#### Human Betaherpesvirus 5

Human Betaherpesvirus 5 or Human Cytomegalovirus (HCMV) belongs to the human herpesvirus family and the betaherpesvirus subfamily and is among the largest viruses known to cause clinical diseases. It is a double-stranded DNA virus with a genome size of around 235 kb and an icosahedral shape measuring from 150 nm to 200 nm in diameter [175]. HCMV was first associated with an infectious disease similar to mononucleosis in healthy individuals in 1965. Currently, it is known to cause a wide range of clinical syndromes, from asymptomatic infection in healthy hosts to severe and even fatal disease in immunocompromised individuals such as transplant recipients [203].

Transmission can occur through direct or indirect contact from person to person with infectious body fluids, including semen, cervical or vaginal secretions, saliva, urine, and blood products. The excretion of HCMV can last for months or years and can be continuous or intermittent. The two main sources of primary HCMV infection during pregnancy are through sexual activity and contact with young children. HCMV can be transmitted from mother to child in utero, intrapartum, or during breastfeeding. History of sexually transmitted diseases, younger sexual onset, and having multiple sexual partners have been associated with seropositivity for HCMV. Horizontal transmission from child to child or from child to adult, particularly in group daycare centers, probably occurs through the transmission of the virus through saliva on hands and toys [204].

CNS cytomegalovirus infection occurs most commonly in patients with severe immunosuppression, such as those with advanced HIV infection (i.e., AIDS) and those who have undergone bone marrow or solid organ transplantation. Immunocompetent patients are affected very rarely. CNS infection can affect the brain (diffuse encephalitis, ventriculoencephalitis, brain mass lesions) or the spinal cord (transverse myelitis, polyradiculomyelitis). HCMV infection of the CNS is less frequent compared to other HCMV infections, both in AIDS patients and in those who have had a solid organ transplant. Studies performed at autopsy have revealed evidence of CNS infection in almost one-third of patients with AIDS [205].

The prevalence of HCMV increases with age. In children aged 1 to 5 years, prevalence can be as low as 20.7% [206], while it approaches 100% in older adults in developing countries. The seroprevalence of HCMV varies according to (i) geographic location, because the highest rates are observed in developing countries; (ii) age, with the rate increasing directly with age; and (iii) socioeconomic level, with higher seroprevalence in countries with unfavorable economic situations and higher population density.

HCMV is most commonly acquired early in life, from childhood to adulthood, through exposure to saliva, tears, urine, feces, breast milk, semen, and other bodily secretions from infected individuals. It can also be transmitted efficiently through organ and tissue transplantation and blood transfusions. Leukoreduction of blood products significantly reduces the risk of transfusion transmitted HCMV infections [203].

The prevalence of HCMV in Brazil is high, but few studies have shown the viral infections of the central nervous system. A study conducted in 2007 showed that among 43 CSF samples, HCMV was detected in 6% [182]. In 2013, an incidence of 7.9% was found in autopsy cases from patients infected with the human immunodeficiency virus (HIV) [207]. However, no association was found between HCMV glycoprotein B (gB) genotypes and HCMV-related central nervous system disease [208]. In 2014, an 18.4% rate of detection of HCMV was reported in patients with suspected central nervous system viral infection in the Western Brazilian Amazon [27].

The genetic polymorphism of envelope glycoproteins, such as the UL55 gene that encodes gB, the UL73 gene that encodes N glycoprotein (gN), and the UL75 gene that encodes H glycoprotein (gH), are used to classify strains of HCMV. Genetic variability has been evaluated in clinical isolates due to its significant role in tropism and tissue virulence that can influence HCMV infectivity or pathogenicity. Currently, there is no consensus on the classification of HCMV strains based on genotype, evolutionary relationship, or clinical relevance [209]. Some authors have hypothesized that the genetic variation among HCMV strains may be the basis for specific clinical manifestations [210,211,212]. HCMV genomes sequenced directly from clinical material using high-throughput sequencing technology reveals variation, multiple-strain infection, recombination, and gene loss [213].

Diagnosis of HCMV-related encephalitis cannot be made in the clinic without the help of images or virological markers. Because patients with CNS HCMV infection have highly variable symptoms, the clinical manifestations suggest brain or spinal cord involvement with rapid or subacute onset, with signs of meningitis in some cases. Infection of the CNS can occur simultaneously in the brain, meninges, spinal cord, and radius as encephalomyelomeningoradiculitis [214]. The main symptoms are diffuse encephalitis, ventriculoencephalitis, brain mass injury that can lead to confusion, reduced concentration, focal signs (hemiparesis, hemianopia), epileptic attacks, nystagmus, ataxia, diplopia or polyradiculomyelitis, necrotizing myelitis with sensory deficits, hypotonic paresis, paraplegia, quadriplegia, urinary retention, and paresthesia [215].

Due to the absence of specific clinical symptomatology, clinical and laboratorial diagnoses via imaging and detection of the virus are important to confirm HCMV infection of the CNS. The method of choice is the detection of HCMV in CSF by qPCR or PCR, and genotypic assays have been evolved to rapidly diagnose drug-resistant viruses [36,216,217]. The main drugs used in the treatment of HCMV act by inhibiting viral DNA synthesis and virostatic: acyclovir (Guanosine analogue), cidofovir (Deoxycytidine analogue), foscarnet (Pyrophosphate analogue), ganciclovir (Acyclic nucleoside analogue of acyclovir), and valganciclovir (nucleoside analog of ganciclovir) [217]. Drug-resistant HCMV should be considered when drugs are used. If the active replication marker, the HCMV pp65 antigen, persists after several weeks of therapy, it may indicate resistant strains. Mutations in the UL97 protein kinase and UL64 DNA polymerase can lead to resistance against ganciclovir, whereas mutations in UL54 can lead to resistance against cidofovir and foscarnet [216]. Resistant/refractory HCMV occurs in a minority of patients, but results in significant morbidity and mortality, and sometimes involves the use of both older antivirals (foscarnet, cidofovir) and novel agents (maribavir, brincidofovir, letermovir) in addition to adjunctive treatments [216]. Rapid genotypic assays can predict resistance to these mutations, assisting alternative treatment in patients who have not responded to primary therapy. Although multiple antiviral agents have efficacy against HCMV, the combination therapy needs to be undertaken with caution to ensure synergy rather than antagonism.

### 2.4. Transmissible Spongiform Encephalopathies or Prion Diseases

Transmissible spongiform encephalopathies (TSEs) are a group of fatal diseases that cause neurodegeneration [218]. Cognitive decline and loss of motor function are the main symptoms of TSEs. The culprit in these diseases is the cellular prion protein (PrP^C^), which is highly abundant in the CNS. PrP^C^ can undergo a conformational change into a form that has the ability to self-associate and become infectious, and is known as prion scrapie protein (PrP^Sc^), the PrP pathogenic form [218].

The history of prion disease began in the 18th century when Merino sheep were found to display symptoms that included gait changes and excessive licking and itching. With time, it was hypothesized to be an infectious disease of viral or bacterial origin [219]. 

The term prion is derived from the acronym for Proteinaceous Infectious Particles and was postulated by Stanley Prusiner in 1982 to define a protein, not a viral particle, that was able to promote infection in the prion diseases. Although these diseases are caused by a single protein without a nucleic acid, their characteristics are similar to those of viral diseases [220].

Currently, it is known that prions cause neurodegenerative damage to humans and several other animals. The prion diseases that affect humans are Kuru, Creutzfeldt-Jakob Disease (CJD), Gerstmann-Straussler-Scheinker disease (GSS), fatal familial insomnia (FFI), and variably protease-sensitive prionopathy (VPSPr) [221]. Bovine spongiform encephalopathy (BSE), also known as mad cow disease, scrapie, and chronic wasting disease, among others, is a form that presents in nonhuman animals [221].

#### 2.4.1. Mechanism of Infection of the Nervous System

TSEs can be transmitted in different ways, including through environmental, intravenous, oral, and ocular routes. Thus, the etiology of prion diseases is classified into sporadic (idiopathic), acquired (infectious and transmitted), and genetic (inherited or familial) [222]. The acquired form occurs mainly by ingesting food products contaminated with PrP^Sc^, through contact with contaminated surgical instruments (iatrogenic), or through using grafts or hormones from cadavers. The acquired forms are considered to account for about 1% of cases in humans [223].

An example of the orally acquired form is the epidemic of Kuru, which occurred in a tribe in Papua New Guinea that had cannibalistic rituals [224]. In addition to Kuru, variant CJD disease (vCJD) is also acquired orally and is related to ingestion of beef contaminated with BSE [225].

**Figure 2:**
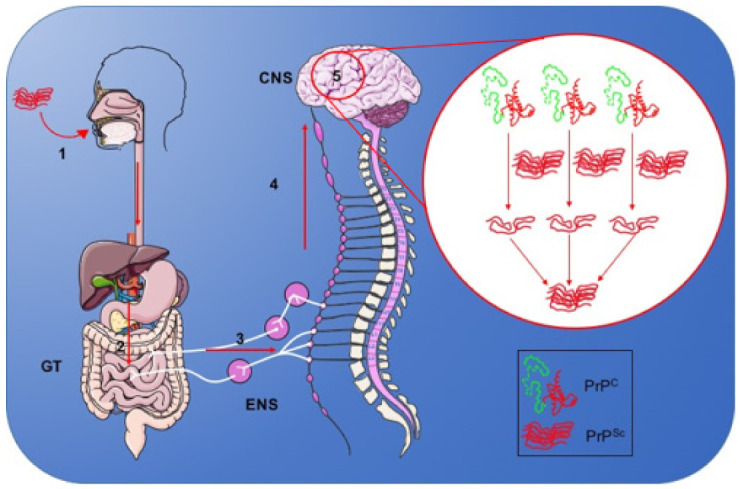
Gut–brain connection in prion infection. In the oral/intragastric route (1) of infection, PrP^Sc^ crosses the gastroin (2), migrates and accumulates in the lymphoid tissues, and infects the enteric nervous system (ENS) (3), propagating to the central nervous system (4). In the brain, PrC^Sc^ interacts with PrP^C^, and, acting as a template, converts it to the pathogenic conformation (5). Conversion follows an autocatalytic replication, forming many copies of PrP^Sc^ that aggregate (5), triggering cell death.

The route of CNS infection for acquired diseases depends on the form of inoculation. In oral/intragastric PrP^Sc^ administration, prions migrate and accumulate in the lymphoid tissues associated with the intestine. Subsequently, other tissues are affected, such as the spleen, tonsils, and lymph nodes. Access to the enteric nervous system (ENS) occurs when nerve cells approach prion-containing lymphoid tissues. After reaching the ENS, there are two pathways that might explain CNS propagation: Through the splanchnic nerve circuit, which involves the celiac ganglion and mesenteric complex, and the parasympathetic vagus nerve circuit (Figure 2) [226]. 

In the sporadic form, the protein conversion occurs spontaneously without known environmental factors. The sporadic form of CJD (sCJD) is the most common TSE in humans, constituting about 85–90% of cases [227]. The genetic form occurs in about 10–15% of cases in humans, in which several penetrating variants in the *PRNP* gene generate a PrP^C^ that is more susceptible to conversion into PrP^Sc^ [227].

#### 2.4.2. Molecular Aspects of Prion Diseases

PrP^C^ is a glycoprotein widely expressed in the CNS, neurons, astrocytes, microglia, and oligodendrocytes, and can also be found in other tissues such as the heart, skeletal muscle, retina, intestine, lungs, testes, and immune cells. PrP^C^ is encoded by the *PRNP* gene and is highly conserved in several species [228]. 

The pathogenesis of prion disease occurs through the conversion of PrP^C^ to PrP^Sc^ (Figure 2). These proteins have no differences in their amino acid sequence, only in their structure. PrP^Sc^ has a greater contribution of secondary structure in the β-sheet (43%) and less in the α-helix (30%) than PrP^C^ [229]. This structural change leads to biochemical changes in PrP^Sc^, such as resistance to proteases, insolubility in nonionic detergents, and the tendency to aggregate [230]. 

The replication of PrP^Sc^ is autocatalytic, and the conversion of PrP^C^ produces new PrP^Sc^ if there is substrate. PrP aggregates sequentially, forming oligomers, protofibrils, and amyloid fibers [231]. Some molecules, such as RNA, glycosaminoglycans, and lipids, act as adjuvant cofactors, facilitating conversion [229,231]. 

The toxicity in neural cells is caused not only by the presence of mature amyloid fibers but also by prefibrillar oligomers that interact in an aberrant manner with different molecules and cellular structures. Among other effects, this leads to activation of apoptosis, mitochondrial dysfunction, oxidative stress, damage and permeabilization of the cell membrane [232]. The main characteristics of TSEs are the presence of accumulated amyloid aggregates in a diffuse manner in synapses and perivascular and perineuronal regions, gliosis, spongiosis, and neuron death [233].

PrP^Sc^ can accumulate in different regions of the brain depending on the type of strain, generating different lesions and clinical features [234]. The specificity of the strain for a given group of cells may be related to the presence of cofactors that help prion replication [234].

#### 2.4.3. Epidemiological Aspects of Prion Diseases

##### Prion Strains

As mentioned above, there are different infectious forms of PrP^Sc^ which are known as prion strains. The different strains are not necessarily related to changes in PrP sequence, because the same sequence can fold differently. Strains are mainly characterized according to differences in disease severity, distribution of PrP aggregates throughout the brain, and incubation times, and are related to heterogeneous structures and physicochemical properties of the aggregates [234].

Prion strains have been mostly characterized in patients with sCJD, the most common prion disease, but very similar patterns are observed in genetic CJD (gCJD), vCJD, iatrogenic CJD (iCJD), sporadic fatal insomnia (sFI), and Kuru. iCJD and Kuru are caused by infection with a sCJD strain [234]. The *PRNP* genotype at the polymorphic codon 129, encoding for a methionine (M) or a valine (V), plays an important role in the disease phenotype. For example, the allele V has been correlated with a plaque-like deposition pattern of PrP^Sc^ [235].

**Figure 3:**
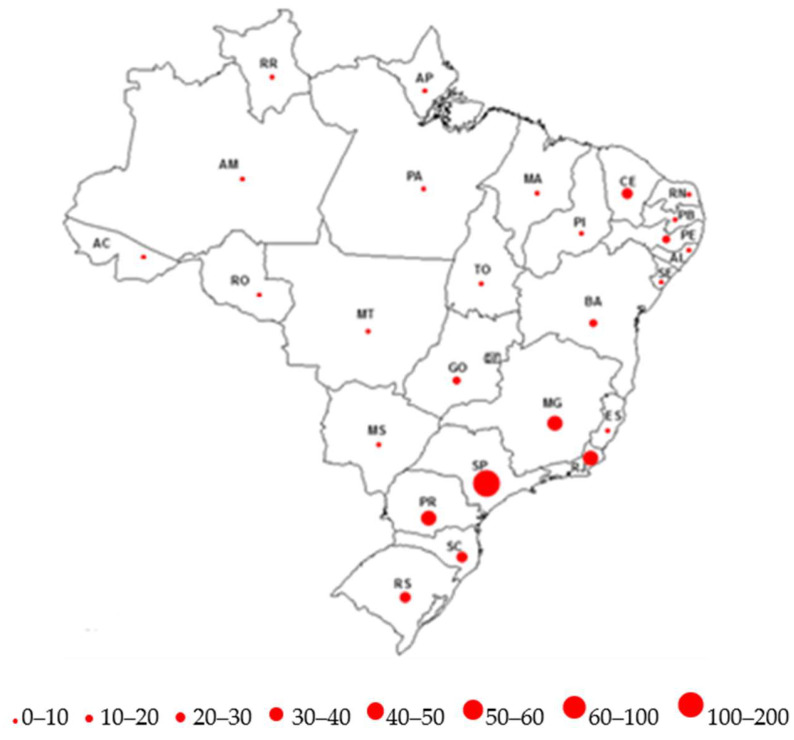
Geographic distribution of confirmed CJD cases in Brazil, 2005–2019.

CJD and its variants have been included on the list of compulsorily reported diseases in Brazil since 2005. The notification organization chart progresses from the identification of the suspected case by the health care services, through its confirmation, until its notification to the local epidemiological surveillance system and registration in the Notifiable Diseases Information System (Sinan) [236]. From 2005 to 2020, 1273 suspected cases of CJD were reported, resulting in the confirmation of 408 cases, with an average age at onset of 62 years of age (Figure 3). No vCJD cases have been reported to date [237]. Previous cases were mainly concentrated in the southeast and south of the country, probably due to the better capability of health care centers in these regions to conduct diagnostic tests.

Few case reports exist of Brazilian patients with prion diseases, and most consist of genetic and iatrogenic forms. The frequency of allele 129 is 46% MM, 48% MV, and 6% VV in the Brazilian population [238], suggesting higher susceptibility for the manifestation of the disease. In addition to the notified cases of sCJD, the following case reports have been published: One family with gCJD T183A mutation [239]; one patient with gCJD V210I mutation and 129M/M [240], one patient with gCJD E200K mutation and 129M/M [241], one patient with gCJD V180I mutation and 129M/M [242], and two probable cases of iCJD due to use of human growth hormone [243,244]. In the case of the very rare GSS disease, the study of two families with the P102L mutation in the *PRNP* gene was reported, representing seven cases in total: Four with 129M/V and two with 129M/M (molecular data was not obtained from one patient) [245].

Although prion diseases have a significant social and economic impact, thus justifying the compulsory reporting in the country, the differential diagnosis among other neurodegenerative diseases is relatively difficult, requiring professional experience and adequate equipment and materials. Thus, the number and geographical distribution of cases in Brazil are underestimated and restricted to major health care centers with the necessary resources and expertise.

#### 2.4.4. Clinical Aspects of CJD Associated with Alterations in the Central Nervous System

Neurodegeneration often leads to cognitive impairment, a very common symptom of diseases such as Alzheimer’s disease (AD), Parkinson’s disease (PD), Huntington’s disease (HD), and CJD. Protein aggregation inside and outside the cell, observed in these pathologies, is thought to be related to the generation of toxic species, leading to death. Classically, prion diseases are called spongiform encephalopathies precisely because the patient’s central nervous tissue displays spongiform change, neuronal loss, and gliosis, all of which can be observed through histological analysis and light microscopy.

CJD evolves rapidly following the onset of symptoms, with a mean survival of about 6 months. The global incidence is 1.5–2 cases per million per year in 70- to 79-year-old people. CJD is classically characterized by symptoms such as ataxia, myoclonus, and cognitive impairment [246]. gCJD and iCJD present clinical aspects similar to those of sCJD. vCJD shows progressive cognitive impairment, ataxia, painful sensory symptoms, myoclonus, and behavioral and psychiatric symptoms (except sleep disorders) [246].

#### 2.4.5. Diagnosis of Prion Diseases

TSEs are diagnosed according to criteria based on clinical evaluation, clinical tests, and biomarkers. Thus, they are classified as possible, probable, and definitive/confirmed. The European Centre for Disease Prevention and Control (ECDC, EU, Solna, Sweden) and the Centers for Disease Control and Prevention (CDC, Atlanta, GA, USA) have the most updated clinical diagnostic criteria. World Health Organization criteria date from 2003. Therefore, they do not include analyses that have improved and developed the criteria after that date [247]. 

The vast majority of definite cases are diagnosed after death, because analyses are made from brain tissue and biopsies are invasive. To classify as a probable or possible TSE, evaluation of symptoms is highly important, but is not sufficient in isolation. Paraclinical tests are essential for the classification of cases and the distinction of other treatable pathologies with similar symptoms [247]. Table 2 indicates the diagnostic criteria for sCJD classification according to the CDC [248].

Two paraclinical tests have gained importance in recent years for improving sensitivity and specificity in diagnosis: The MRI (with sequences such as diffusion-weighted imaging (DWI), fluid-attenuated inversion recovery (FLAIR), apparent diffusion coefficient (ADC)) and the real-time quaking-induced conversion assay (RT-QuIC). MRI shows 80% sensitivity and specificity for detecting CJD [249]. RT-QuIC shows 100% sensitivity and specificity for sCJD, combining the analysis of CSF and olfactory mucosa (OM) [250]. 

In addition to its high sensitivity and specificity, the RT-QuIC assay has several advantages, not only for sCJD but also for other TSEs [247]. It is a relatively simple method based on the auto-propagating properties of PrP^Sc^. A positive RT-QuIC test, together with neuropsychiatric symptoms, is considered sufficient to classify the case as probable CJD [248]. In the future, with a larger number of studies certifying the accuracy of this test, it may even become a sufficient tool to define CJD and other TSE cases. 

Brazilian guidelines for TSE diagnosis are still based on the WHO protocol [251]. Recently, RT-QuIC was implemented at the Federal University of Rio de Janeiro, and samples from Brazilian patients were evaluated. The test proved to be important to diagnose cases of patients with atypical clinical presentation, in addition to blindly diagnose known cases [252]. The study also demonstrated the possibility of carrying out this type of test in Brazil and, therefore, its inclusion in the notification and investigation protocols.

#### 2.4.6. Treatment of Prion Diseases

Prion diseases are still untreatable today. There is no curative or preventive treatment for these disorders, and only palliative treatments exist to attempt to mitigate some symptoms and provide supportive care (such as antipsychotics, antidepressants, and anticonvulsants). The rarity of the disease and the absence of pre-symptomatic diagnosis also hinder the development of efficient therapies.

Some molecules with interesting effects in vitro and in animal models have been tested in human clinical trials, but without success. Observational studies were clinically tested with pentosan polysulfate, quinacrine, flupirtine, and doxycycline without significant effects [253]. These studies are challenging because variables such as the stage of the disease, speed of clinical evolution, number of patients including a placebo group, and distribution of allele 129 genotype in the studied group are important to limit the observed effects. Many other molecules with different structures are being tested in vitro and in vivo, showing promising results in inhibiting protein conversion, aggregation, and cell disfunction. These molecules may be used in future clinical trials [253]. 

Another therapeutic approach is to decrease PrP^C^ expression, leading to a decrease in substrate for conversion. Antisense oligonucleotides (ASOs) are molecules able to specifically bind in a complementary manner, degrade the target messenger RNA, and, consequently, decrease protein production. This approach is now in preclinical development for prion diseases [254]. 

Immunotherapy is also a promising approach against prion disease because it may block PrP^Sc^ and/or PrP^C^, hindering the conversion. Protective effects of active immunization studies have not proven sufficient to date [255]. Passive immunization has had more promising results. A humanized anti-PrP^C^ monoclonal antibody, called PRN100, is being tested in six patients with sCJD, but the results of this trial are not yet known. Some antibodies show an important effect in prolonging the survival of prion-infected mice models [255]. 

The use of cell therapies has been suggested as a strategy to recover the damaged areas of the affected brain. Grafted neural stem cells (NSCs) may support the survival of existing neurons [256] and stimulate endogenous NSCs [257]. However, NCSs are susceptible to PrP^Sc^ infection and replication [258]. Moreover, the ability to propagate between cells and consequent transmission to grafted cells may make the treatment unfeasible. The use of NCSs from PrP knockout mice before the onset of clinical signs has been shown to lead to an increase in incubation and survival times [259]. These studies have shown promise for the use of NSCs in the treatment of TSEs, but additional studies will need to be carried out to ensure their effectiveness and safety. In conclusion, diagnosis and therapy must evolve together so that such devastating diseases are detected and treated early, increasing the quality of life of patients and their families.

### 2.5. SARS-CoV-2

In the viewpoint of the authors, it is worth mentioning the recently recognized infection caused by SARS-CoV-2 (severe acute respiratory syndrome coronavirus 2) in the CNS infection context. Coronavirus disease 2019 (COVID-19) is caused by SARS-CoV-2 infection, and the clinical symptoms range from mild to severe, for which acute respiratory distress is observed. Although several studies have demonstrated that SARS-CoV-2 can be associated with CNS infections such as meningitis, encephalitis, and Guillain-Barre syndrome [260], further investigations must be conducted to understand putative associations between SARS-CoV-2 and CNS infection because the virus is poorly detected in CSF [261]. In Brazil, information about the SARS-CoV-2 involvement in CNS infection is still scarce, and few reports have suggested a possible association with neurological disease [261,262].

## 3. Concluding Remarks

Viral CNS infections have been identified as a significant cause of morbidity affecting millions of children and adults, particularly in low- and middle-income countries. These infections may be associated not only with motor problems, but also with cognitive, behavioral, and mental sequelae. Additionally, viral CNS infections can trigger neurodegenerative disorders such as Parkinson’s disease, multiple sclerosis, and Alzheimer’s disease [6,8,9,10]. Because viral CNS infections are a public health concern, there should be greater awareness among public managers and health systems of the need to define policies and strategies to improve the monitoring of these infections and to support patients with sequelae. As a result of writing this review to assess the spectrum of neurological diseases associated with viral infection in Brazil, we realized the extent to which CNS-associated diseases have been neglected. 

Based on this scenario, we can suggest several ways to reduce the impact of these infections, especially in developing countries: (i) Strengthening surveillance systems to identify the emergence of novel neuro-invasive viruses; (ii) ameliorating the tools for early diagnosis from radiographic evidence, and molecular tests to avoid diagnostic mistakes and delays in specific therapy; (iii) performing long-term epidemiological studies to evaluate the incidence and prevalence of the most common CNS infections; and, finally, (iv) implementing effective rehabilitation programs to support patients with sequelae.

## Figures and Tables

**Table 1:** Viral and prion infections in CNS-associated infections in Brazil.

Genus/Causative Agents	Structure	Transmission	Route to Brain	Neurological Disorders	Types Commonly CNS-Associated Infections
Enterovirus	Single-stranded RNA Nonenveloped Icosahedral symmetry	Fecal–oral, respiratory	Infected endothelial cells; retrograde axonal transport; Trojan Horse within infected monocytes	Aseptic meningitis, acute flaccid paralysis, encephalitis/meningoencephalitis	E6, E7, E11, E18, E30, CVB5, CVA2, EV-A71, CVB5, E6, E7, E11, CVA13, EV-C99, CVB2, E6, E18, E30
Flavivirus	Single-stranded RNA Enveloped Icosahedral symmetry	Via arthropod vectors	Hematogenous; axonal transport; Trojan Horse within infected leukocytes	Encephalitis, microencephaly (Zika)	Dengue, Yellow fever, Zika
Alphavirus	Single-stranded RNA Enveloped Icosahedral symmetry	Via arthropod vectors	Anterograde axonal transport	Encephalitis	Chikungunya
Simplexvirus	Double-stranded DNA Enveloped Icosahedral symmetry	Oral-to-oral, oral–genital contact with sores	Primary infections: olfactory, hematogenous and genital (HSV-2), retrograde axonal transport Reactivation: from neurons trigeminal or sacral (HSV-2) ganglia, anterograde transport to reach CNS	Encephalitis, meningoencephalitis	HSV-1 HSV-2
Varicellovirus	Double-stranded DNA Enveloped Icosahedral symmetry	Via droplets, aerosol, direct contact	Peripheral spread VZV- reactivation from sensory ganglia- central spread to brain or to temporal arteritis or to spinal cord arteries	Meningoencephalitis, myelitis, cranial neuropathies	VZV
Cytomegalovirus	Double-stranded DNA Enveloped Icosahedral symmetry	Contact with saliva or urine; sexual; contact breast milk; transplanted organs; blood transfusions	Hematopoietic cells monocytes—systemic spread- brain, spinal cord, meninges, nerve roots	Encephalitis, myelitis, polyradiculopathy, multifocal neuropathy	CMV
Prion *scrapie*	Beta structure enriched form of cellular prion protein	Iatrogenic, oral, blood	Infected epithelium, lymphoid tissues	Rapidly progressive cognitive impairment with behavioral and visual disturbances, ataxia, and myoclonus.	BSE, vCJD, iCJD

**Table 2:** ECDC and CDC current guidelines for sCJD diagnosis.

sCJD Diagnostic	Signals and Symptoms
Possible	Progressive dementia AND at least 2 out of the following 4 clinical features: Myoclonus, cerebellar or visual disturbance, pyramidal and extrapyramidal dysfunction, akinetic mutism; AND the absence of a positive result for any of the 4 tests that would classify a case as “probable”; AND duration of illness less than 2 years; AND without routine investigations indicating an alternative diagnosis.
Probable	Neuropsychiatric disorder plus positive RT-QuIC in cerebrospinal fluid (CSF) or other tissues OR rapidly progressive dementia; AND at least 2 out of the following 4 clinical features: Myoclonus, cerebellar or visual disorder, pyramidal and extrapyramidal dysfunction, akinetic mutism; AND a positive result on at least 1 of the following laboratory tests: A typical EEG (periodic sharp wave complexes) during an illness of any duration, a positive 14-3-3 CSF assay in patients with a disease duration of less than 2 years, high signal in caudate/putamen on magnetic resonance imaging (MRI) brain scan or at least 2 cortical regions (temporal, parietal, occipital) either on diffusion-weighted imaging (DWI) or fluid-attenuated inversion recovery (FLAIR); AND without routine investigations indicating an alternative diagnosis.
Definite	Neuropathological confirmation and/or confirmation of PrP^res^ by immunocytochemistry or Western blot and/or presence of scrapie fibers.

## Data Availability

Not applicable.

## References

[1] Muñoz L.S., Garcia M.A., Gordon-Lipkin E., Parra B., Pardo C.A. (2018). Emerging viral infections and their impact on the global burden of neurological disease. Semin. Neurol..

[2] Robertson F.C., Lepard J.R., Mekary R.A., Davis M.C., Yunusa I., Gormley W.B., Baticulon R.E., Mahmud M.R., Misra B.K., Rattani A. (2018). Epidemiology of central nervous system infectious diseases: A meta-analysis and systematic review with implications for neurosurgeons worldwide. J. Neurosurg..

[3] Jones K.E., Patel N.G., Levy M.A., Storeygard A., Balk D., Gittleman J.L., Daszak P. (2008). Global trends in emerging infectious diseases. Nature.

[4] Harapan H., Itoh N., Yufika A., Winardi W., Keam S., Te H., Megawati D., Hayati Z., Wagner A.L., Mudatsir M. (2020). Coronavirus disease 2019 (COVID-19): A literature review. J. Infect. Public Health.

[5] Olival K.J., Daszak P. (2005). The ecology of emerging neurotropic viruses. J. Neurovirol..

[6] Clé M., Eldin P., Briant L., Lannuzel A., Simonin Y., van de Perre P., Cabié A., Salinas S. (2020). Neurocognitive impacts of arbovirus infections. J. Neuroinflammation.

[7] Dalman C., Allebeck P., Gunnell D., Harrison G., Kristensson K., Lewis G., Lofving S., Rasmussen F., Wicks S., Karlsson H. (2008). Infections in the CNS during childhood and the risk of subsequent psychotic illness: A cohort study of more than one million Swedish subjects. Am. J. Psychiatry.

[8] Naughton S.X., Raval U., Pasinetti G.M. (2020). The viral hypothesis in Alzheimer’s disease: Novel insights and pathogen-based biomarkers. J. Pers. Med..

[9] Alfahad T., Nath A. (2013). Retroviruses and amyotrophic lateral sclerosis. Antivir. Res..

[10] Wouk J., Rechenchoski D.Z., Rodrigues B.C.D., Ribelato E.V., Faccin-Galhardi L.C. (2021). Viral infections and their relationship to neurological disorders. Arch. Virol..

[11] Carrillo-Salinas F.J., Mestre L., Mecha M., Feliú A., del Campo R., Villarrubia N., Espejo C., Montalbán X., Álvarez-Cermeño J.C., Villar L.M. (2017). Gut dysbiosis and neuroimmune responses to brain infection with Theiler’s murine encephalomyelitis virus. Sci. Rep..

[12] Li N., Ma W.T., Pang M., Fan Q.L., Hua J.L. (2019). The commensal microbiota and viral infection: A comprehensive review. Front. Immunol..

[13] Yarandi S.S., Peterson D.A., Treisman G.J., Moran T.H., Pasricha P.J. (2016). Modulatory effects of gut microbiota on the central nervous system: How gut could play a role in neuropsychiatric health and diseases. J. Neurogastroenterol. Motil..

[14] Tetz G., Tetz V. (2018). Prion-like domains in eukaryotic viruses. Sci. Rep..

[15] Peters C.E., Carette J.E. (2021). Return of the neurotropic enteroviruses: Co-opting cellular pathways for infection. Viruses.

[16] Tapparel C., Siegrist F., Petty T.J., Kaiser L. (2013). Picornavirus and enterovirus diversity with associated human diseases. Infect. Genet. Evol..

[17] Chen B.S., Lee H.C., Lee K.M., Gong Y.N., Shih S.R. (2020). Enterovirus and encephalitis. Front. Microbiol..

[18] Majer A., McGreevy A., Booth T.F. (2020). Molecular pathogenicity of enteroviruses causing neurological disease. Front. Microbiol..

[19] Hasbun R., Rosenthal N., Balada-Llasat J.M., Chung J., Duff S., Bozzette S., Zimmer L., Ginocchio C.C. (2017). Epidemiology of meningitis and encephalitis in the United States, 2011–2014. Clin. Infect. Dis..

[20] Posnakoglou L., Tatsi E.-B., Chatzichristou P., Siahanidou T., Kanaka-Gantenbein C., Syriopoulou V., Michos A. (2021). Molecular epidemiology of enterovirus in children with central nervous system infections. Viruses.

[21] Suresh S., Forgie S., Robinson J. (2018). Non-polio enterovirus detection with acute flaccid paralysis: A systematic review. J. Med. Virol..

[22] Cassemiro K.M.S.M., Burlandy F.M., Silva E.E. (2016). Rare natural type 3/type 2 intertypic capsid recombinant vaccine-related poliovirus isolated from a case of acute flaccid paralysis in Brazil, 2015. J. Gen. Virol..

[23] Ramalho E., Sousa I., Burlandy F., Costa E., Dias A., Serrano R., Oliveira M., Lopes R., Debur M., Burger M. (2019). Identification and phylogenetic characterization of human enteroviruses isolated from cases of aseptic meningitis in Brazil, 2013–2017. Viruses.

[24] Sousa I.P., Oliveira M.L.A., Burlandy F.M., Machado R.S., Oliveira S.S., Tavares F.N., Gomes-Neto F., da Costa E.V., da Silva E.E. (2020). Molecular characterization and epidemiological aspects of non-polio enteroviruses isolated from acute flaccid paralysis in Brazil: A historical series (2005–2017). Emerg. Microbes Infect..

[25] Compagnoli-Carmona R.C., Caetano-Machado B., Aparecida de Sousa C., Vieira H.R., Moraes Alves M.R., Farias de Souza K.A., de Souza-Gregório D., Costa-Vilanova B., Sampaio-Tavares-Timenetsky M.D.C. (2020). Distribution of species enterovirus B in patients with central nervous system infections in São Paulo State, Brazil. J. Med. Virol..

[26] Gomes M.d.L., de Castro C.M., Oliveira M.J., da Silva E.E. (2002). Neutralizing antibodies to enterovirus 71 in Belém, Brazil. Mem. Inst. Oswaldo Cruz.

[27] Bastos M.S., Lessa N., Naveca F.G., Monte R.L., Braga W.S., Figueiredo L.T., Ramasawmy R., Mourão M.P. (2014). Detection of Herpesvirus, Enterovirus, and Arbovirus infection in patients with suspected central nervous system viral infection in the Western Brazilian Amazon. J. Med. Virol..

[28] De Oliveira D.B., Candiani T.M., Franco-Luiz A.P.M., Almeida G.M.F., Abrahão J.S., Rios M., Coimbra R.S., Kroon E.G. (2017). Etiological agents of viral meningitis in children from a dengue-endemic area, Southeast region of Brazil. J. Neurol. Sci..

[29] Suresh S., Rawlinson W.D., Andrews P.I., Stelzer-Braid S. (2020). Global epidemiology of nonpolio enteroviruses causing severe neurological complications: A systematic review and meta-analysis. Rev. Med. Virol..

[30] Dos Santos G.P.L., Skraba I., Oliveira D., Lima A.A.F., de Melo M.M.M., Kmetzsch C.I., Costa E.V., da Silva E.E. (2006). Enterovirus meningitis in Brazil, 1998–2003. J. Med. Virol..

[31] Pinto-Junior V.L., Rebelo M.C., Costa E.V., Silva E.E., Bóia M.N. (2009). Description of a widespread outbreak of aseptic meningitis due to echovirus 30 in Rio de Janeiro state, Brazil. Braz. J. Infect. Dis..

[32] Luchs A., Russo D.H., Cilli A., Costa F.F., Morillo S.G., Machado B.C., Pellini A.C., Carmona R.C.C., Timenetsky M.C. (2008). Echovirus 6 associated to aseptic meningitis outbreak, in São Joaquim da Barra, São Paulo, Brazil. Braz. J. Microbiol..

[33] Kmetzsch C.I., Balkie E.M., Monteiro A., Costa E.V., dos Santos G.P., da Silva E.E. (2006). Echovirus 13 aseptic meningitis, Brazil. Emerg. Infect. Dis..

[34] Fernandez-Garcia M.D., Kebe O., Fall A.D., Ndiaye K. (2017). Identification and molecular characterization of non-polio enteroviruses from children with acute flaccid paralysis in West Africa, 2013–2014. Sci. Rep..

[35] Tseng F.-C., Huang H.-C., Chi C.-Y., Lin T.-L., Liu C.-C., Jian J.-W., Hsu L.-C., Wu H.-S., Yang J.-Y., Chang Y.-W. (2007). Epidemiological survey of enterovirus infections occurring in Taiwan between 2000 and 2005: Analysis of sentinel physician surveillance data. J. Med. Virol..

[36] Costa B.K.D., Sato D.K. (2020). Viral encephalitis: A practical review on diagnostic approach and treatment. J. Pediatr..

[37] Li M.L., Chen B.S., Shih S.R. (2020). Editorial: Viral encephalitis. Front. Microbiol..

[38] Valle D.A.D., Santos M.L.S.F., Giamberardino H.I.G., Raboni S.M., Scola R.H. (2020). Acute childhood viral encephalitis in southern Brazil. Pediatric Infect. Dis. J..

[39] Sousa I.P., Machado R.S., Burlandy F.M., Silva E.E.D. (2020). Detection and characterization of a coxsackievirus B2 strain associated with acute meningoencephalitis, Brazil, 2018. Rev. Soc. Bras. Med. Trop..

[40] WHO (2015). Enterovirus Surveillance Guidelines—Guidelines for Enterovirus Surveillance in Support of the Polio Eradication.

[41] Wilder-Smith A., Gubler D.J., Weaver S.C., Monath T.P., Heymann D.L., Scott T.W. (2017). Epidemic arboviral diseases: Priorities for research and public health. Lancet Infect. Dis..

[42] Harapan H., Michie A., Sasmono R.T., Imrie A. (2020). Dengue: A minireview. Viruses.

[43] Vieira M.A.D.C., Costa C.H.N., Linhares A.D.C., Borba A.S., Henriques D.F., Silva E.V.P.D., Tavares F.N., Batista F.M.A., Guimarães H.C.L., Martins L.C. (2018). Potential role of dengue virus, chikungunya virus and Zika virus in neurological diseases. Mem. Inst. Oswaldo Cruz.

[44] Ministério da Saúde (2017). Manual de Vigilância Sentinela de Doenças Neuroinvasivas por Arbovírus.

[45] Vasilakis N., Weaver S.C. (2017). Flavivirus transmission focusing on Zika. Curr. Opin. Virol..

[46] Kuhn R.J., Zhang W., Rossmann M.G., Pletnev S.V., Corver J., Lenches E., Jones C.T., Mukhopadhyay S., Chipman P.R., Strauss E.G. (2002). Structure of dengue virus: Implications for flavivirus organization, maturation, and fusion. Cell.

[47] Heinz F.X., Stiasny K. (2012). Flaviviruses and flavivirus vaccines. Vaccine.

[48] Gould E.A., Solomon T. (2008). Pathogenic flaviviruses. Lancet.

[49] Lindquist L., Vapalahti O. (2008). Tick-borne encephalitis. Lancet.

[50] Samy A.M., Alkishe A.A., Thomas S.M., Wang L., Zhang W. (2018). Mapping the potential distributions of etiological agent, vectors, and reservoirs of Japanese Encephalitis in Asia and Australia. Acta Trop..

[51] Melo A.S., Aguiar R.S., Amorim M.M., Arruda M.B., Melo F.O., Ribeiro S.T., Batista A.G., Ferreira T., dos Santos M.P., Sampaio V.V. (2016). Congenital Zika virus infection: Beyond neonatal microcephaly. JAMA Neurol..

[52] Monath T.P., Barrett A.D. (2003). Pathogenesis and pathophysiology of yellow fever. Adv. Virus Res..

[53] Beck A.S., Barrett A.D. (2015). Current status and future prospects of yellow fever vaccines. Expert Rev. Vaccines.

[54] Screaton G., Mongkolsapaya J., Yacoub S., Roberts C. (2015). New insights into the immunopathology and control of dengue virus infection. Nat. Rev. Immunol..

[55] Li G.H., Ning Z.J., Liu Y.M., Li X.H. (2017). Neurological manifestations of dengue infection. Front. Cell. Infect. Microbiol..

[56] Mustafá Y.M., Meuren L.M., Coelho S.V.A., de Arruda L.B. (2019). Pathways exploited by flaviviruses to counteract the blood-brain barrier and invade the central nervous system. Front. Microbiol..

[57] Mehta R., Soares C.N., Medialdea-Carrera R., Ellul M., da Silva M.T.T., Rosala-Hallas A., Jardim M.R., Burnside G., Pamplona L., Bhojak M. (2018). The spectrum of neurological disease associated with Zika and chikungunya viruses in adults in Rio de Janeiro, Brazil: A case series. PLoS Negl. Trop. Dis..

[58] Verma S., Kumar M., Gurjav U., Lum S., Nerurkar V.R. (2010). Reversal junction proteins degradation by matrix metalloproteinases inhibitor. Virology.

[59] Jurado K.A., Yockey L.J., Wong P.W., Lee S., Huttner A.J., Iwasaki A. (2018). Antiviral CD8 T cells induce Zika-virus-associated paralysis in mice. Nat. Microbiol..

[60] Wang K., Wang H., Lou W., Ma L., Li Y., Zhang N., Wang C., Li F., Awais M., Cao S. (2018). IP-10 promotes blood-brain barrier damage by inducing tumor necrosis factor alpha production in Japanese encephalitis. Front. Immunol..

[61] Hussmann K.L., Samuel M.A., Kim K.S., Diamond M.S., Fredericksen B.L. (2013). Differential replication of pathogenic and nonpathogenic strains of West Nile virus within astrocytes. J. Virol..

[62] Garcez P.P., Loiola E.C., Madeiro da Costa R., Higa L.M., Trindade P., Delvecchio R., Nascimento J.M., Brindeiro R., Tanuri A., Rehen S.K. (2016). Zika virus impairs growth in human neurospheres and brain organoids. Science.

[63] Li F., Wang Y., Yu L., Cao S., Wang K., Yuan J., Wang C., Cui M., Fu Z.F. (2015). Viral infection of the central nervous system and neuroinflammation precede blood-brain barrier disruption during Japanese encephalitis virus infection. J. Virol..

[64] Salomão N.G., Rabelo K., Póvoa T.F., Alves A.M.B., da Costa S.M., Gonçalves A.J.S., Amorim J.F., Azevedo A.S., Nunes P.C.G., Basílio-de-Oliveira C.A. (2018). BALB/c mice infected with DENV-2 strain 66985 by the intravenous route display injury in the central nervous system. Sci. Rep..

[65] Marinho P.E.S., Kroon E.G. (2019). Flaviviruses as agents of childhood central nervous system infections in Brazil. New Microbes New Infect..

[66] Chen R., Vasilakis N. (2011). Dengue—Quo tu et quo vadis?. Viruses.

[67] Weaver S.C., Vasilakis N. (2009). Molecular evolution of dengue viruses: Contributions of phylogenetics to understanding the history and epidemiology of the preeminent arboviral disease. Infect. Genet. Evol..

[68] Guzman M.G., Harris E. (2015). Dengue. Lancet.

[69] Solomon T., Dung N.M., Vaughn D.W., Kneen R., Thao L.T., Raengsakulrach B., Loan H.T., Day N.P., Farrar J., Myint K.S. (2000). Neurological manifestations of dengue infection. Lancet.

[70] Carod-Artal F.J., Wichmann O., Farrar J., Gascón J. (2013). Neurological complications of dengue virus infection. Lancet Neurol..

[71] Gupta M., Nayak R., Khwaja G.A., Chowdhury D. (2013). Acute disseminated encephalomyelitis associated with dengue infection: A case report with literature review. J. Neurol. Sci..

[72] Mello C.D.S., Cabral-Castro M.J., Silva de Faria L.C., Peralta J.M., Puccioni-Sohler M. (2020). Dengue and chikungunya infection in neurologic disorders from endemic areas in Brazil. Neurol. Clin. Pract..

[73] Soares C.N., Cabral-Castro M.J., Peralta J.M., de Freitas M.R., Zalis M., Puccioni-Sohler M. (2011). Review of the etiologies of viral meningitis and encephalitis in a dengue endemic region. J. Neurol. Sci..

[74] Baldaçara L., Ferreira J.R., Filho L.C., Venturini R.R., Coutinho O.M., Camarço W.C., Fernandes C.C., Júnior E.V. (2013). Behavior disorder after encephalitis caused by dengue. J. Neuropsychiatry Clin. Neurosci..

[75] Madi D., Achappa B., Ramapuram J.T., Chowta N., Laxman M., Mahalingam S. (2014). Dengue encephalitis—A rare manifestation of dengue fever. Asian Pac. J. Trop. Biomed..

[76] Mathew T., Badachi S., Sarma G.R., Nadig R. (2015). “Dot sign” in dengue encephalitis. Ann. Indian Acad. Neurol..

[77] Thisyakorn U., Thisyakorn C., Limpitikul W., Nisalak A. (1999). Dengue infection with central nervous system manifestations. Southeast Asian J. Trop. Med. Public Health.

[78] Misra U., Kalita J., Syam U., Dhole T. (2006). Neurological manifestations of dengue virus infection. J. Neurol. Sci..

[79] Liou L., Lan S., Lai C. (2008). Electroencephalography burst suppression in a patient with dengue encephalopathy: A case report. Clin. Neurophysiol..

[80] Oehler E., le Hénaff O., Ghawche F. (2012). Neurological manifestations of dengue. Presse Med..

[81] Soares C., Faria L., Peralta J., de Freitas M., Puccioni-Sohler M. (2006). Dengue infection: Neurological manifestations and cerebrospinal fluid (CSF) analysis. J. Neurol. Sci..

[82] Araújo F., Nogueira R., Araújo Me S., Perdigão A., Cavalcanti L., Brilhante R., Rocha M., Vilar D.F., Holanda S.S., Braga M. (2012). Dengue in patients with central nervous system manifestations, Brazil. Emerg. Infect. Dis..

[83] Marinho P.E., Bretas de Oliveira D., Candiani T.M., Crispim A.P., Alvarenga P.P., Castro F.C., Abrahão J.S., Rios M., Coimbra R.S., Kroon E.G. (2017). Meningitis associated with simultaneous infection by multiple dengue virus serotypes in children, Brazil. Emerg. Infect. Dis..

[84] Liou L., Lan S., Lai C. (2008). Dengue fever with ischemic stroke: A case report. Neurologist.

[85] Verma R., Sahu R., Holla V. (2014). Neurological manifestations of dengue infection: A review. J. Neurol. Sci..

[86] Vargas-Sánchez A., Chiquete E., Gutiérrez-Plascencia P., Castañeda-Moreno V., Alfaro-Castellanos D., Paredes-Casillas P., Ruiz-Sandovala J.L. (2014). Cerebellar hemorrhage in a patient during the convalescent phase of dengue fever. J. Stroke.

[87] Kunishige M., Mitsui T., Tan B., Leong H., Takasaki T., Kurane I., Mihara A., Matsumoto T. (2004). Preferential gray matter involvement in dengue myelitis. Neurology.

[88] Chanthamat N., Sathirapanya P. (2010). Acute transverse myelitis associated with dengue viral infection. J. Spinal Cord Med..

[89] Weeratunga P.N., Caldera M.C., Gooneratne I.K., Gamage R., Perera P. (2014). Neurological manifestations of dengue: A cross sectional study. Travel Med. Infect. Dis..

[90] Fong C.Y., Hlaing C.S., Tay C.G., Kadir K.A., Goh K.J., Ong L.C. (2016). Longitudinal extensive transverse myelitis with cervical epidural haematoma following dengue virus infection. Eur. J. Paediatr. Neurol..

[91] Mota M.T., Estofolete C.F., Zini N., Terzian A.C., Gongora D.V., Maia I.L., Nogueira M.L. (2017). Transverse myelitis as an unusual complication of dengue fever. Am. J. Trop. Med. Hyg..

[92] Sharma C.M., Kumawat B.L., Ralot T., Tripathi G., Dixit S. (2011). Guillain-Barre syndrome occurring during dengue fever. J. Indian Med. Assoc..

[93] Jain R.S., Handa R., Prakash S., Nagpal K., Gupta P. (2014). Acute hypokalemic quadriparesis: An atypical neurological manifestation of dengue virus. J. Neurovirol..

[94] Langerak T., van Rooij I., Doornekamp L., Chandler F., Baptista M., Yang H., Koopmans M.P.G., GeurtsvanKessel C.H., Jacobs B.C., Rockx B. (2021). Guillain-Barré syndrome in Suriname; clinical presentation and identification of preceding infections. Front. Neurol..

[95] Grijalva I., Grajales-Muñiz C., González-Bonilla C., Borja-Aburto V.H., Paredes-Cruz M., Guerrero-Cantera J., González-Ibarra J., Vallejos-Parás A., Rojas-Mendoza T., Santacruz-Tinoco C.E. (2020). Zika and dengue but not chikungunya are associated with Guillain-Barré syndrome in Mexico: A case-control study. PLoS Negl. Trop. Dis..

[96] Prateek S.V., Paliwal N., Tak H. (2019). Dengue, Guillain-Barré syndrome, and cerebral infarction: A case of rare complication. Indian J. Crit. Care Med..

[97] Asbury A.K., Cornblath D.R. (1990). Assessment of current diagnostic criteria for Guillain-Barré syndrome. Ann. Neurol..

[98] Shahrizaila N., Lehmann H.C., Kuwabara S. (2021). Guillain-Barré syndrome. Lancet.

[99] Dick G.W., Kitchen S.F., Haddow A.J. (1952). Zika virus. I. Isolations and serological specificity. Trans. R. Soc. Trop. Med. Hyg..

[100] Heymann D.L., Hodgson A., Sall A.A., Freedman D.O., Staples J.E., Althabe F., Baruah K., Mahmud G., Kandun N., Vasconcelos P.F. (2016). Zika virus and microcephaly: Why is this situation a PHEIC?. Lancet.

[101] Lanciotti R.S., Kosoy O.L., Laven J.J., Velez J.O., Lambert A.J., Johnson A.J., Stanfield S.M., Duffy M.R. (2008). Genetic and serologic properties of Zika virus associated with an epidemic, Yap State, Micronesia, 2007. Emerg. Infect. Dis..

[102] Freitas D.A., Souza-Santos R., Carvalho L.M.A., Barros W.B., Neves L.M., Brasil P., Wakimoto M.D. (2020). Congenital Zika syndrome: A systematic review. PLoS ONE.

[103] Duffy M.R., Chen T.H., Hancock W.T., Powers A.M., Kool J.L., Lanciotti R.S., Pretrick M., Marfel M., Holzbauer S., Dubray C. (2009). Zika virus outbreak on Yap Island, Federated States of Micronesia. N. Engl. J. Med..

[104] Zammarchi L., Tappe D., Fortuna C., Remoli M.E., Günther S., Venturi G., Bartoloni A., Schmidt-Chanasit J. (2015). Zika virus infection in a traveller returning to Europe from Brazil, March 2015. Eurosurveillance.

[105] Brasil P., Calvet G.A., Siqueira A.M., Wakimoto M., de Sequeira P.C., Nobre A., Quintana M.e.S., Mendonça M.C., Lupi O., de Souza R.V. (2016). Zika virus outbreak in Rio de Janeiro, Brazil: Clinical characterization, epidemiological and virological aspects. PLoS Negl. Trop. Dis..

[106] Oehler E., Watrin L., Larre P., Leparc-Goffart I., Lastere S., Valour F., Baudouin L., Mallet H., Musso D., Ghawche F. (2014). Zika virus infection complicated by Guillain-Barre syndrome—Case report, French Polynesia, December 2013. Eurosurveillance.

[107] Cao-Lormeau V.M., Blake A., Mons S., Lastère S., Roche C., Vanhomwegen J., Dub T., Baudouin L., Teissier A., Larre P. (2016). Guillain-Barré syndrome outbreak associated with Zika virus infection in French Polynesia: A case-control study. Lancet.

[108] Leonhard S.E., Halstead S., Lant S.B., Militão de Albuquerque M.F.P., de Brito C.A.A., de Albuquerque L.B.B., Ellul M.A., de Oliveira França R.F., Gourlay D., Griffiths M.J. (2021). Guillain-Barré syndrome during the Zika virus outbreak in Northeast Brazil: An observational cohort study. J. Neurol. Sci..

[109] Brasil P., Sequeira P.C., Freitas A.D., Zogbi H.E., Calvet G.A., de Souza R.V., Siqueira A.M., de Mendonca M.C., Nogueira R.M., de Filippis A.M. (2016). Guillain-Barré syndrome associated with Zika virus infection. Lancet.

[110] Peixoto H.M., Romero G.A.S., de Araújo W.N., de Oliveira M.R.F. (2019). Guillain-Barré syndrome associated with Zika virus infection in Brazil: A cost-of-illness study. Trans. R. Soc. Trop. Med. Hyg..

[111] Brito Ferreira M.L., Antunes de Brito C.A., Moreira Á., de Morais Machado M., Henriques-Souza A., Cordeiro M.T., de Azevedo Marques E.T., Pena L.J. (2017). Guillain-Barré syndrome, acute disseminated encephalomyelitis and encephalitis associated with Zika virus infection in Brazil: Detection of viral RNA and isolation of virus during late infection. Am. J. Trop. Med. Hyg..

[112] Muñoz L.S., Parra B., Pardo C.A. (2017). Study NEitA: Neurological implications of Zika virus infection in adults. J. Infect. Dis..

[113] Da Silva I.R.F., Frontera J.A., Bispo de Filippis A.M., Nascimento O.J.M.D. (2017). Group R-G-ZR: Neurologic complications associated with the Zika virus in Brazilian adults. JAMA Neurol..

[114] Mécharles S., Herrmann C., Poullain P., Tran T.H., Deschamps N., Mathon G., Landais A., Breurec S., Lannuzel A. (2016). Acute myelitis due to Zika virus infection. Lancet.

[115] Carteaux G., Maquart M., Bedet A., Contou D., Brugières P., Fourati S., Cleret de Langavant L., de Broucker T., Brun-Buisson C., Leparc-Goffart I. (2016). Zika virus associated with meningoencephalitis. N. Engl. J. Med..

[116] Rozé B., Najioullah F., Fergé J.L., Dorléans F., Apetse K., Barnay J.L., Daudens-Vaysse E., Brouste Y., Césaire R., Fagour L. (2017). Guillain-Barré syndrome associated with Zika virus infection in Martinique in 2016: A prospective study. Clin. Infect. Dis..

[117] Roth W., Tyshkov C., Thakur K., Vargas W. (2017). Encephalomyelitis following definitive Zika virus infection. Neurol. Neuroimmunol. Neuroinflamm..

[118] Douam F., Ploss A. (2018). Yellow fever virus: Knowledge gaps impeding the fight against an old foe. Trends Microbiol..

[119] Vasconcelos P.F. (2003). Yellow fever. Rev. Soc. Bras. Med. Trop..

[120] Possas C., Lourenço-de-Oliveira R., Tauil P.L., Pinheiro F.P., Pissinatti A., Cunha R.V.D., Freire M., Martins R.M., Homma A. (2018). Yellow fever outbreak in Brazil: The puzzle of rapid viral spread and challenges for immunisation. Mem. Inst. Oswaldo Cruz.

[121] Giovanetti M., de Mendonça M.C.L., Fonseca V., Mares-Guia M.A., Fabri A., Xavier J., de Jesus J.G., Gräf T., dos Santos Rodrigues C.D., dos Santos C.C. (2019). Yellow fever virus reemergence and spread in southeast Brazil, 2016–2019. J. Virol..

[122] Rezende I.M., Sacchetto L., Munhoz de Mello É., Alves P.A., Iani F.C.M., Adelino T.R., Duarte M.M., Cury A.L.F., Bernardes A.F.L., Santos T.A. (2018). Persistence of Yellow fever virus outside the Amazon Basin, causing epidemics in southeast Brazil, from 2016 to 2018. PLoS Negl. Trop. Dis..

[123] Cunha M.S., da Costa A.C., de Azevedo Fernandes N.C.C., Guerra J.M., dos Santos F.C.P., Nogueira J.S., D’Agostino L.G., Komninakis S.V., Witkin S.S., Ressio R.A. (2019). Epizootics due to Yellow fever virus in São Paulo State, Brazil: Viral dissemination to new areas (2016–2017). Sci. Rep..

[124] Sacchetto L., Drumond B.P., Han B.A., Nogueira M.L., Vasilakis N. (2020). Re-emergence of yellow fever in the neotropics—Quo vadis?. Emerg. Top. Life Sci..

[125] De Oliveira Figueiredo P., Stoffella-Dutra A.G., Barbosa Costa G., Silva de Oliveira J., Dourado Amaral C., Duarte Santos J., Soares Rocha K.L., Araújo Júnior J.P., Lacerda Nogueira M., Zazá Borges M.A. (2020). Re-emergence of yellow fever in Brazil during 2016–2019: Challenges, lessons learned, and perspectives. Viruses.

[126] Bryant J.E., Holmes E.C., Barrett A.D. (2007). Out of Africa: A molecular perspective on the introduction of yellow fever virus into the Americas. PLoS Pathog..

[127] Delatorre E., de Abreu F.V.S., Ribeiro I.P., Gómez M.M., dos Santos A.A.C., Ferreira-de-Brito A., Neves M.S.A.S., Bonelly I., de Miranda R.M., Furtado N.D. (2019). Distinct YFV lineages co-circulated in the central-western and southeastern Brazilian regions from 2015 to 2018. Front. Microbiol..

[128] Paules C.I., Fauci A.S. (2017). Yellow fever—Once again on the radar screen in the Americas. N. Engl. J. Med..

[129] Jones E.M., Wilson D.C. (1972). Clinical features of yellow fever cases at Vom Christian Hospital during the 1969 epidemic on the Jos Plateau, Nigeria. Bull. World Health Organ..

[130] Martin M., Tsai T.F., Cropp B., Chang G.J., Holmes D.A., Tseng J., Shieh W., Zaki S.R., Al-Sanouri I., Cutrona A.F. (2001). Fever and multisystem organ failure associated with 17D-204 yellow fever vaccination: A report of four cases. Lancet.

[131] Vasconcelos P.F., Luna E.J., Galler R., Silva L.J., Coimbra T.L., Barros V.L., Monath T.P., Rodigues S.G., Laval C., Costa Z.G. (2001). Serious adverse events associated with yellow fever 17DD vaccine in Brazil: A report of two cases. Lancet.

[132] Kengsakul K., Sathirapongsasuti K., Punyagupta S. (2002). Fatal myeloencephalitis following yellow fever vaccination in a case with HIV infection. J. Med. Assoc Thail..

[133] Gardner C.L., Ryman K.D. (2010). Yellow fever: A reemerging threat. Clin. Lab. Med..

[134] Lara A.N., Miyaji K.T., Ibrahim K.Y., Lopes M.H., Sartori A.M.C. (2021). Adverse events following yellow fever vaccination in immunocompromised persons. Rev. Inst. Med. Trop. Sao Paulo.

[135] Juan-Giner A., Kimathi D., Grantz K.H., Hamaluba M., Kazooba P., Njuguna P., Fall G., Dia M., Bob N.S., Monath T.P. (2021). Immunogenicity and safety of fractional doses of yellow fever vaccines: A randomised, double-blind, non-inferiority trial. Lancet.

[136] Martins R.M., Pavão A.L., de Oliveira P.M., dos Santos P.R., Carvalho S.M., Mohrdieck R., Fernandes A.R., Sato H.K., de Figueiredo P.M., von Doellinger V.R. (2014). Adverse events following yellow fever immunization: Report and analysis of 67 neurological cases in Brazil. Vaccine.

[137] De Menezes Martins R., Fernandes Leal M.L., Homma A. (2015). Serious adverse events associated with yellow fever vaccine. Hum. Vaccines Immunother..

[138] Barnett E.D. (2007). Yellow fever: Epidemiology and prevention. Clin. Infect. Dis..

[139] Oliveira H.S.B., Araujo P.P., Sousa J.R.P., Donis A.C.G., Moreira D., Makssoudian A. (2020). Serious adverse event: Late neurotropic disease associated with yellow fever vaccine. Einstein.

[140] Venkatesan A., Geocadin R.G. (2014). Diagnosis and management of acute encephalitis: A practical approach. Neurol. Clin. Pract..

[141] Arboviral Diseases, Neuroinvasive and Non-Neuroinvasive 2015 Case Definition. wwwn.cdc.gov/nndss/conditions/arboviral-diseases-neuroinvasive-and-nonneuroinvasive/case-definition/2015/.

[142] Tyler K.L., Roos K.L. (2017). The expanding spectrum of Zika virus infections of the nervous system. JAMA Neurol..

[143] Silva L.A., Dermody T.S. (2017). Chikungunya virus: Epidemiology, replication, disease mechanisms, and prospective intervention strategies. J. Clin. Investig..

[144] Baxter V.K., Heise M.T. (2020). Immunopathogenesis of alphaviruses. Adv. Virus Res..

[145] Zeller H., van Bortel W., Sudre B. (2016). Chikungunya: Its history in Africa and Asia and its spread to new regions in 2013–2014. J. Infect. Dis..

[146] Nunes M.R., Faria N.R., de Vasconcelos J.M., Golding N., Kraemer M.U., de Oliveira L.F., Azevedo R.S., da Silva D.E., da Silva E.V., da Silva S.P. (2015). Emergence and potential for spread of Chikungunya virus in Brazil. BMC Med..

[147] De Souza T.M.A., Ribeiro E.D., Corrêa V.C.E., Damasco P.V., Santos C.C., de Bruycker-Nogueira F., Chouin-Carneiro T., Faria N.R.D.C., Nunes P.C.G., Heringer M. (2018). Following in the footsteps of the Chikungunya virus in Brazil: The first autochthonous cases in Amapá in 2014 and its emergence in Rio de Janeiro during 2016. Viruses.

[148] Lopes N., Nozawa C., Linhares R.E.C. (2014). Características gerais e epidemiologia dos arbovírus emergentes no Brasil. Inst. Evandro Chagas Pará Rev. Pan-Amaz. Saude.

[149] Weaver S.C., Lecuit M. (2015). Chikungunya virus and the global spread of a mosquito-borne disease. N. Engl. J. Med..

[150] Bandeira A.C., Campos G.S., Sardi S.I., Rocha V.F., Rocha G.C. (2016). Neonatal encephalitis due to Chikungunya vertical transmission: First report in Brazil. IDCases.

[151] Mehta R., Gerardin P., de Brito C.A.A., Soares C.N., Ferreira M.L.B., Solomon T. (2018). The neurological complications of chikungunya virus: A systematic review. Rev. Med. Virol..

[152] Corrêa D.G., di Maio Ferreira F.C.P.A., Hygino da Cruz L.C., Brasil P., Rueda Lopes F.C. (2020). Longitudinal brain magnetic resonance imaging of children with perinatal Chikungunya encephalitis. Neuroradiol. J..

[153] Lima S.T.S., Souza W.M., Cavalcante J.W., da Silva Candido D., Fumagalli M.J., Carrera J.P., Simões Mello L.M., de Carvalho Araújo F.M., Cavalcante Ramalho I.L., de Almeida Barreto F.K. (2020). Fatal outcome of chikungunya virus infection in Brazil. Clin. Infect. Dis..

[154] Chatterjee S.N., Chakravarti S.K., Mitra A.C., Sarkar J.K. (1965). Virological investigation of cases with neurological complications during the outbreak of haemorrhagic fever in Calcutta. J. Indian Med. Assoc..

[155] Thiruvengadam K.V., Kalyanasundaram V., Rajgopal J. (1965). Clinical and pathological studies on chikungunya fever in Madras city. Indian J. Med. Res..

[156] Couderc T., Chrétien F., Schilte C., Disson O., Brigitte M., Guivel-Benhassine F., Touret Y., Barau G., Cayet N., Schuffenecker I. (2008). A mouse model for Chikungunya: Young age and inefficient type-I interferon signaling are risk factors for severe disease. PLoS Pathog..

[157] Fraisier C., Koraka P., Belghazi M., Bakli M., Granjeaud S., Pophillat M., Lim S.M., Osterhaus A., Martina B., Camoin L. (2014). Kinetic analysis of mouse brain proteome alterations following Chikungunya virus infection before and after appearance of clinical symptoms. PLoS ONE.

[158] Passoni G., Langevin C., Palha N., Mounce B.C., Briolat V., Affaticati P., De Job E., Joly J.S., Vignuzzi M., Saleh M.C. (2017). Imaging of viral neuroinvasion in the zebrafish reveals that Sindbis and chikungunya viruses favour different entry routes. Dis. Models Mech..

[159] Economopoulou A., Dominguez M., Helynck B., Sissoko D., Wichmann O., Quenel P., Germonneau P., Quatresous I. (2009). Atypical Chikungunya virus infections: Clinical manifestations, mortality and risk factors for severe disease during the 2005–2006 outbreak on Réunion. Epidemiol. Infect..

[160] Tandale B.V., Sathe P.S., Arankalle V.A., Wadia R.S., Kulkarni R., Shah S.V., Shah S.K., Sheth J.K., Sudeep A.B., Tripathy A.S. (2009). Systemic involvements and fatalities during Chikungunya epidemic in India, 2006. J. Clin. Virol..

[161] Kashyap R.S., Morey S., Bhullar S., Baheti N., Chandak N., Purohit H., Taori G., Daginawala H. (2014). Determination of Toll-like receptor-induced cytokine profiles in the blood and cerebrospinal fluid of Chikungunya patients. Neuroimmunomodulation.

[162] Azevedo M.B., Coutinho M.S.C., Silva M.A.D., Arduini D.B., Lima J.D.V., Monteiro R., Mendes B.N.B., Lemos M.C.F., Noronha C.P., Saraceni V. (2018). Neurologic manifestations in emerging arboviral diseases in Rio de Janeiro City, Brazil, 2015–2016. Rev. Soc. Bras. Med. Trop..

[163] Martins H.A., Bernardino S.N., Santos C.C., Ribas V.R. (2016). Chikungunya and myositis: A case report in Brazil. J. Clin. Diagn. Res..

[164] Rocha V.F.D., de Oliveira A.H.P., Bandeira A.C., Sardi S.I., Garcia R.F., Magalhães S.A., Sampaio C.A., Campos Soares G. (2018). Chikungunya virus infection associated with encephalitis and anterior uveitis. Ocul. Immunol. Inflamm..

[165] Sá P.K.O., Nunes M.M., Leite I.R., Campelo M.D.G.L., Leão C.F.R., Souza J.R., Castellano L.R., Fernandes A.I.V. (2017). Chikungunya virus infection with severe neurologic manifestations: Report of four fatal cases. Rev. Soc. Bras. Med. Trop..

[166] Brito Ferreira M.L., Militão de Albuquerque M.F.P., de Brito C.A.A., de Oliveira França R.F., Porto Moreira Á., de Morais Machado M., da Paz Melo R., Medialdea-Carrera R., Dornelas Mesquita S., Lopes Santos M. (2020). Neurological disease in adults with Zika and chikungunya virus infection in Northeast Brazil: A prospective observational study. Lancet Neurol..

[167] Soares D.S., Fortaleza L.Y., Melo M.C. (2020). Chikungunya-induced manic episode in a patient with no psychiatric history: A case report. Braz. J. Psychiatry.

[168] Pereira L.P., Villas-Bôas R., Scott S.S.O., Nóbrega P.R., Sobreira-Neto M.A., Castro J.D.V., Cavalcante B., Braga-Neto P. (2017). Encephalitis associated with the chikungunya epidemic outbreak in Brazil: Report of 2 cases with neuroimaging findings. Rev. Soc. Bras. Med. Trop..

[169] Puccioni-Sohler M., Farias L.C., Cabral-Castro M.J., Zalis M.G., Kalil R.S., Salgado M.C.F. (2018). Cerebrospinal fluid immunoglobulins as potential biomarkers of Chikungunya encephalitis. Emerg. Infect. Dis..

[170] Scott S.S.O., Braga-Neto P., Pereira L.P., Nóbrega P.R., de Assis Aquino Gondim F., Sobreira-Neto M.A., Schiavon C.C.M. (2017). Immunoglobulin-responsive chikungunya encephalitis: Two case reports. J. Neurovirol..

[171] Silva N.M., Santos N.C., Martins I.C. (2020). Dengue and Zika viruses: Epidemiological history, potential therapies, and promising vaccines. Trop. Med. Infect. Dis..

[172] Alice F. (1956). Infecção humana pelo vírus “leste” da encefalite equina. Bol. Inst. Biol. Bahia.

[173] Casseb A.R., Brito T.C., Silva M.R.M., Chiang J.O., Martins L.C., Silva S.P., Henriques D.F., Casseb L.M.N., Vasconcelos P.F.C. (2016). Prevalence of antibodies to equine alphaviruses in the State of Pará, Brazil. Arq. Inst. Biol..

[174] Da Costa V.G., de Rezende Féres V.C., Saivish M.V., de Lima Gimaque J.B., Moreli M.L. (2017). Silent emergence of Mayaro and Oropouche viruses in humans in Central Brazil. Int. J. Infect. Dis..

[175] International Committee on Taxonomy of Viruses Executive Committee (2020). The new scope of virus taxonomy: Partitioning the virosphere into 15 hierarchical ranks. Nat. Microbiol..

[176] Kawada J.I. (2018). Neurological disorders associated with human alphaherpesviruses. Adv. Exp. Med. Biol..

[177] Gnann J.W., Whitley R.J. (2017). Herpes simplex encephalitis: An update. Curr Infect Dis Rep..

[178] Levitz R.E. (1998). Herpes simplex encephalitis: A review. Heart Lung.

[179] Whitley R.J. (2015). Herpes simplex virus infections of the central nervous system. Continuum (Minneap Minn).

[180] Duarte L.F., Farías M.A., Álvarez D.M., Bueno S.M., Riedel C.A., González P.A. (2019). Herpes simplex virus type 1 infection of the central nervous system: Insights into proposed interrelationships with neurodegenerative disorders. Front. Cell. Neurosci..

[181] Nagel M.A., Niemeyer C.S., Bubak A.N. (2020). Central nervous system infections produced by varicella zoster virus. Curr. Opin. Infect. Dis..

[182] Mendoza L.P., Bronzoni R.V., Takayanagui O.M., Aquino V.H., Figueiredo L.T. (2007). Viral infections of the central nervous system in Brazil. J. Infect..

[183] Pfaff F., Groth M., Sauerbrei A., Zell R. (2016). Genotyping of herpes simplex virus type 1 by whole-genome sequencing. J. Gen. Virol..

[184] Kolb A.W., Adams M., Cabot E.L., Craven M., Brandt C.R. (2011). Multiplex sequencing of seven ocular herpes simplex virus type-1 genomes: Phylogeny, sequence variability, and SNP distribution. Investig. Ophthalmol. Vis. Sci..

[185] Szpara M.L., Gatherer D., Ochoa A., Greenbaum B., Dolan A., Bowden R.J., Enquist L.W., Legendre M., Davison A.J. (2014). Evolution and diversity in human herpes simplex virus genomes. J. Virol..

[186] Parsons L.R., Tafuri Y.R., Shreve J.T., Bowen C.D., Shipley M.M., Enquist L.W., Szpara M.L. (2015). Rapid genome assembly and comparison decode intrastrain variation in human alphaherpesviruses. mBio.

[187] Newman R.M., Lamers S.L., Weiner B., Ray S.C., Colgrove R.C., Diaz F., Jing L., Wang K., Saif S., Young S. (2015). Genome sequencing and analysis of geographically diverse clinical isolates of herpes simplex virus 2. J. Virol..

[188] Zell R., Taudien S., Pfaff F., Wutzler P., Platzer M., Sauerbrei A. (2012). Sequencing of 21 varicella-zoster virus genomes reveals two novel genotypes and evidence of recombination. J. Virol..

[189] Kolb A.W., Ané C., Brandt C.R. (2013). Using HSV-1 genome phylogenetics to track past human migrations. PLoS ONE.

[190] Piret J., Boivin G. (2016). Antiviral resistance in herpes simplex virus and varicella-zoster virus infections: Diagnosis and management. Curr. Opin. Infect. Dis..

[191] Corey L., Whitley R.J., Stone E.F., Mohan K. (1988). Difference between herpes simplex virus type 1 and type 2 neonatal encephalitis in neurological outcome. Lancet.

[192] Raschilas F., Wolf M., Delatour F., Chaffaut C., De Broucker T., Chevret S., Lebon P., Canton P., Rozenberg F. (2002). Outcome of and prognostic factors for herpes simplex encephalitis in adult patients: Results of a multicenter study. Clin. Infect. Dis..

[193] Solomon T., Michael B.D., Smith P.E., Sanderson F., Davies N.W., Hart I.J., Holland M., Easton A., Buckley C., Kneen R. (2012). Management of suspected viral encephalitis in adults—Association of British Neurologists and British Infection Association National Guidelines. J. Infect..

[194] Schleede L., Bueter W., Baumgartner-Sigl S., Opladen T., Weigt-Usinger K., Stephan S., Smitka M., Leiz S., Kaiser O., Kraus V. (2013). Pediatric herpes simplex virus encephalitis: A retrospective multicenter experience. J. Child Neurol..

[195] Aurelius E., Johansson B., Skoldenberg B., Staland A., Forsgren M. (1991). Rapid diagnosis of herpes simplex encephalitis by nested polymerase chain reaction assay of cerebrospinal fluid. Lancet.

[196] Bhullar S.S., Chandak N.H., Purohit H.J., Taori G.M., Daginawala H.F., Kashyap R.S. (2014). Determination of viral load by quantitative real-time PCR in herpes simplex encephalitis patients. Intervirology.

[197] Poissy J., Champenois K., Dewilde A., Melliez H., Georges H., Senneville E., Yazdanpanah Y. (2012). Impact of herpes sim- plex virus load and red blood cells in cerebrospinal fluid upon herpes simplex meningo-encephalitis outcome. BMC Infect. Dis..

[198] Bhullar S.S., Chandak N.H., Baheti N.N., Purohit H.J., Taori G.M., Daginawala H.F., Kashyap R.S. (2016). Diagnosis of herpes simplex encephalitis by ELISA using antipeptide antibodies against type-common epitopes of glycoprotein B of herpes simplex viruses. J. Immunoass. Immunochem..

[199] Skoldenberg B., Forsgren M., Alestig K., Forkman A., Lövgren K., Norrby R., Stiernstedt G., Forsgren M., Bergström T., Dahlqvist E. (1984). Acyclovir versus vidarabine in herpes simplex encephalitis. Randomised multicentre study in consecutive Swedish patients. Lancet.

[200] Whitley R.J., Alford C.A., Hirsch M.S., Schooley R.T., Luby J.P., Aoki F.Y., Hanley D., Nahmias A.J., Soong S.-J., The NIAID Collaborative Antiviral Study Group (1986). Vidarabine versus acyclovir therapy in herpes simplex encephalitis. N. Engl. J. Med..

[201] Elbers J.M., Bitnun A., Richardson S.E., Ford-Jones E.L., Tellier R., Wald R.M., Petric M., Kolski H., Heurter H., MacGregor D. (2007). A 12-year prospective study of childhood herpes simplex encephalitis: Is there a broader spectrum of disease?. Pediatrics.

[202] Aksamit A.J. (2021). Treatment of viral encephalitis. Neurol. Clin..

[203] Dioverti M.V., Razonable R.R. (2016). Cytomegalovirus. Microbiol. Spectr..

[204] Davis N.L., King C.C., Kourtis A.P. (2017). Cytomegalovirus infection in pregnancy. Birth Defects Res..

[205] Bowen L.N., Smith B., Reich D., Quezado M., Nath A. (2016). HIV-associated opportunistic CNS infections: Pathophysiology, diagnosis and treatment. Nat. Rev. Neurol..

[206] Petersen M.R., Patel E.U., Abraham A.G., Quinn T.C., Tobian A.A.R. (2020). Changes in cytomegalovirus seroprevalence among U.S. children aged 1 to 5 years: The national health and nutrition examination surveys. Clin. Infect. Dis..

[207] Chimelli L., Rosemberg S., Hahn M.D., Lopes M.B., Netto M.B. (1992). Pathology of the central nervous system in patients infected with the human immunodeficiency virus (HIV): A report of 252 autopsy cases from Brazil. Neuropathol. Appl. Neurobiol..

[208] Vilas Boas L.S., de Souza V.A., Penalva de Oliveira A.C., Rodriguez Viso A.T., Nascimento Filho A.M., Nascimento M.C., Pannuti C.S. (2003). Cytomegalovirus glycoprotein B genotypes and central nervous system disease in AIDS patients. J. Med. Virol..

[209] Martí-Carreras J., Maes P. (2019). Human cytomegalovirus genomics and transcriptomics through the lens of next-generation sequencing: Revision and future challenges. Virus Genes.

[210] Ross S.A., Pati P., Jensen T.L., Goll J.B., Gelber C.E., Singh A., McNeal M., Boppana S.B., Bernstein D.I. (2020). Cytomegalovirus genetic diversity following primary infection. J. Infect. Dis..

[211] Barrado L., Prieto C., Hernando S., Folgueira L. (2018). Detection of glycoproteins B and H geno- types to predict the development of Cytomegalovirus disease in solid organ transplant recipients. J. Clin. Virol..

[212] Hu H., Peng W., Peng Q., Cheng Y. (2020). Cytomegalovirus genotype distribution among congenital and perinatal infected patients with HCMV-associated thrombocytopenia. Fetal Pediatric Pathol..

[213] Suárez N.M., Wilkie G.S., Hage E., Camiolo S., Holton M., Hughes J., Maabar M., Vattipally S.B., Dhingra A., Gompels U.A. (2019). Human cytomegalovirus genomes sequenced directly from clinical material: Variation, multiple-strain infection, recombination, and gene loss. J. Infect. Dis..

[214] Lüttmann S., Husstedt I.W., Lügering N., Heese C., Stoll R., Domschke W., Evers S., Kuchelmeister K., Gullotta F. (1997). Cytomegalovirus encephalomyelomeningoradiculitis in acquired immunodeficiency syndrome (AIDS). J. Infect..

[215] Kawasaki H., Kosugi I., Meguro S., Iwashita T. (2017). Pathogenesis of developmental anomalies of the central nervous system induced by congenital cytomegalovirus infection. Pathol. Int..

[216] Maschke M., Kastrup O., Diener H.C. (2002). CNS manifestations of cytomegalovirus infections: Diagnosis and treatment. CNS Drugs.

[217] Bookstaver P.B., Mohorn P.L., Shah A., Tesh L.D., Quidley A.M., Kothari R., Bland C.M., Weissman S. (2017). Management of viral central nervous system infections: A primer for clinicians. J. Cent. Nerv. Syst. Dis..

[218] Scheckel C., Aguzzi A. (2018). Prions, prionoids and protein misfolding disorders. Nat. Rev. Genet..

[219] Zabel M.D., Reid C. (2015). A brief history of prions. Pathog. Dis..

[220] Prusiner S.B. (1982). Novel proteinaceous infectious particles cause scrapie. Science.

[221] Imran M., Mahmood S. (2011). An overview of animal prion diseases. Virol. J..

[222] Hamaguchi T., Sakai K., Kobayashi A., Kitamoto T., Ae R., Nakamura Y., Sanjo N., Arai K., Koide M., Katada F. (2020). Characterization of sporadic Creutzfeldt-Jakob disease and history of neurosurgery to identify potential iatrogenic cases. Emerg. Infect. Dis..

[223] Brown P., Brandel J.-P., Sato T., Nakamura Y., MacKenzie J., Will R.G., Ladogana A., Pocchiari M., Leschek E.W., Schonberger L.B. (2012). Iatrogenic Creutzfeldt-Jakob disease, final assessment. Emerg. Infect. Dis..

[224] Collinge J., Whitfield J., McKintosh E., Beck J., Mead S., Thomas D.J., Alpers M.P. (2006). Kuru in the 21st century—An acquired human prion disease with very long incubation periods. Lancet.

[225] Will R.G., Ironside J.W., Zeidler M., Cousens S.N., Estibeiro K., Alperovitch A., Poser S., Pocchiari M., Hofman A., Smith P.G. (1996). A new variant of Creutzfeldt-Jakob disease in the UK. Lancet.

[226] Sisó S., González L., Jeffrey M. (2010). Neuroinvasion in prion diseases: The roles of ascending neural infection and blood dissemination. Interdiscip. Perspect. Infect. Dis..

[227] Minikel E.V., Vallabh S.M., Lek M., Estrada K., Samocha K.E., Sathirapongsasuti J.F., McLean C.Y., Tung J.Y., Yu L.P.C., Gambetti P. (2016). Quantifying prion disease penetrance using large population control cohorts. Sci. Transl. Med..

[228] Schätzl H.M., da Costa M., Taylor L., Cohen F.E., Prusiner S.B. (1995). Prion protein gene variation among primates. J. Mol. Biol..

[229] Silva J.L., Gomes M.P.B.M.P.B., Vieira T.C.R.G., Cordeiro Y. (2010). PrP interactions with nucleic acids and glycosaminoglycans in function and disease. Front. Biosci..

[230] Sengupta I., Udgaonkar J.B. (2018). Structural mechanisms of oligomer and amyloid fibril formation by the prion protein. Chem. Commun..

[231] Deleault N.R., Piro J.R., Walsh D.J., Wang F., Ma J., Geoghegan J.C., Supattapone S. (2012). Isolation of phosphatidylethanolamine as a solitary cofactor for prion formation in the absence of nucleic acids. Proc. Natl. Acad. Sci. USA.

[232] Taneja V., Verma M., Vats A. (2015). Toxic species in amyloid disorders: Oligomers or mature fibrils. Ann. Indian Acad. Neurol..

[233] Solforosi L., Milani M., Mancini N., Clementi M., Burioni R. (2013). A closer look at prion strains. Prion.

[234] Rossi M., Baiardi S., Parchi P. (2019). Understanding prion strains: Evidence from studies of the disease forms affecting humans. Viruses.

[235] Miyazono M., Kitamoto T., Doh-Ura K., Iwaki T., Tateishi J. (1992). Creutzfeldt-Jakob disease with codon 129 polymorphism (Valine): A comparative study of patients with codon 102 point mutation or without mutations. Acta Neuropathol..

[236] Gattás V.L., Lima Neto A.S., Dimech G.S., Mancini D., Cantarino L.M., Marins J.R.P., Luna E.J.A. (2007). New variant of Creutzfeldt-Jakob (vCJD) disease and other human prion diseases under epidemiological surveillance in Brazil. Dement. Neuropsychol..

[237] Da Cunha J.E.G. DCJ EpiShiny. https://epicjd.shinyapps.io/dcjBRASIL/.

[238] De Paula E.V., Addas-Carvalho M., Costa D.S.P., Saad S.T.O., Gilli S.C.O. (2005). Genotype frequencies at codon 129 of the Prion Protein Gene in Brazil: Implications in susceptibility to variant Creutzfeldt-Jakob disease compared to European and Asian populations. Eur. J. Epidemiol..

[239] Nitrini R., Rosemberg S., Passos-Bueno M.R., Teixeira Da Silva L.S., Iughetti P., Papadopoulos M., Carrilho P.M., Caramelli P., Albrecht S., Zatz M. (1997). Familial spongiform encephalopathy associated with a novel prion protein gene mutation. Ann. Neurol..

[240] Huang N., Marie S.K.N., Kok F., Nitrini R. (2001). Familial Creutzfeldt-Jakob disease associated with a point mutation at codon 210 of the prion protein gene. Arq. Neuropsiquiatr..

[241] Smid J., Martins V.R., Landemberger M.C., Riva D., Anghinah R., Nitrini R. (2007). Creutzfeldt-Jakob disease associated with a missense mutation at codon 200 of the prion protein gene in Brazil. Dement. Neuropsychol..

[242] De Souza R.K.M., Josviak N.D., Batistela M.S., Santos P.S.F., Landemberger M.C., Ramina R. (2017). First case of V180I rare mutation in a Brazilian patient with Creutzfeldt-Jakob disease. Prion.

[243] Ferreira Caboclo L.O.S., Huang N., Lepski G.A., Livramento J.A., Buchpiguel C.A., Porto C.S., Nitrini R. (2002). Iatrogenic Creutzfeldt-Jakob disease following human growth hormone therapy: Case report. Arq. Neuropsiquiatr..

[244] Macario M.E., Moura-Neto V., Vaisman M., Araujo H.M.M., Buescu A., Cordeiro J.G.H., Chagas C. (1992). Abnormal proteins in the cerebrospinal fluid of a patient with Creutzfeldt-Jakob disease following administration of human pituitary growth hormone. Braz. J. Med. Biol. Res..

[245] Smid J., Neto A.S., Landemberger M.C., Machado C.F., Nóbrega P.R., Canedo N.H.S., Schultz R.R., Naslavsky M.S., Rosemberg S., Kok F. (2017). High phenotypic variability in gerstmann-sträussler-scheinker disease. Arq. Neuropsiquiatr..

[246] Fragoso D.C., Gonçalves Filho A.L.d.M., Pacheco F.T., Barros B.R., Littig I.A., Nunes R.H., Maia Júnior A.C.M., da Rocha A.J. (2017). Imaging of Creutzfeldt-Jakob disease: Imaging patterns and their differential diagnosis. Radiographics.

[247] Ascari L.M., Rocha S.C., Gonçalves P.B., Vieira T.C.R.G., Cordeiro Y. (2020). Challenges and advances in antemortem diagnosis of human transmissible spongiform encephalopathies. Front. Bioeng. Biotechnol..

[248] CDC’s Diagnostic Criteria for Creutzfeldt-Jakob Disease (CJD). https://www.cdc.gov/prions/cjd/diagnostic-criteria.html.

[249] Gibson L.M., Chappell F.M., Summers D., Collie D.A., Sellar R., Best J., Knight R., Ironside J.W., Wardlaw J.M. (2018). Post-mortem magnetic resonance imaging in patients with suspected prion disease: Pathological confirmation, sensitivity, specificity and observer reliability. A national registry. PLoS ONE.

[250] Fiorini M., Iselle G., Perra D., Bongianni M., Capaldi S., Sacchetto L., Ferrari S., Mombello A., Vascellari S., Testi S. (2020). High diagnostic accuracy of RT-QuIC assay in a prospective study of patients with suspected sCJD. Int. J. Mol. Sci..

[251] Brown P., Brunk C., Budka H., Cervenakova L., Collie D., Green A., Ironside J., Knight R., MacKenzie J., Pergami P. (2003). WHO Manual for Surveillance of Human Transmissible Spongiform Encephalopathies, Including Variant Creutzfeldt-Jakob Disease.

[252] Barbosa B.J.A.P., Castrillo B.B., Alvim R.P., de Brito M.H., Gomes H.R., Brucki S.M.D., Smid J., Nitrini R., Landemberger M.C., Martins V.R. (2020). Second-generation RT-QuIC assay for the diagnosis of Creutzfeldt-Jakob disease patients in Brazil. Front. Bioeng. Biotechnol..

[253] Mead S., Tagliavini F. (2018). Clinical trials. Handb. Clin. Neurol..

[254] Raymond G.J., Zhao H.T., Race B., Raymond L.D., Williams K., Swayze E.E., Graffam S., Le J., Caron T., Stathopoulos J. (2019). Antisense oligonucleotides extend survival of prion-infected mice. JCI Insight.

[255] Ma Y., Ma J. (2020). Immunotherapy against prion disease. Pathogens.

[256] Chen B., Gao X.Q., Yang C.X., Tan S.K., Sun Z.L., Yan N.H., Pang Y.G., Yuan M., Chen G.J., Xu G.T. (2009). Neuroprotective effect of grafting GDNF gene-modified neural stem cells on cerebral ischemia in rats. Brain Res..

[257] Frid K., Binyamin O., Fainstein N., Keller G., Ben-Hur T., Gabizon R. (2018). Autologous neural progenitor cell transplantation into newborn mice modeling for E200K genetic prion disease delays disease progression. Neurobiol. Aging.

[258] Relaño-Ginès A., Gabelle A., Hamela C., Belondrade M., Casanova D., Mourton-Gilles C., Lehmann S., Crozet C. (2013). Prion replication occurs in endogenous adult neural stem cells and alters their neuronal fate: Involvement of endogenous neural stem cells in prion diseases. PLoS Pathog..

[259] Relaño-Ginés A., Lehmann S., Bencsik A., Herva M.E., Torres J.M., Crozet C.A. (2011). Stem cell therapy extends incubation and survival time in prion-infected mice in a time window-dependant manner. J. Infect. Dis..

[260] Yavarpour-Bali H., Ghasemi-Kasman M. (2020). Update on neurological manifestations of COVID-19. Life Sci..

[261] Espíndola O.M., Brandão C.O., Gomes Y.C.P., Siqueira M., Soares C.N., Lima M.A.S.D., Leite A.C.C.B., Torezani G., Araujo A.Q.C., Silva M.T.T. (2021). Cerebrospinal fluid findings in neurological diseases associated with COVID-19 and insights into mechanisms of disease development. Int. J. Infect. Dis..

[262] Domingues R.B., Mendes-Correa M.C., de Moura Leite F.B.V., Sabino E.C., Salarini D.Z., Claro I., Santos D.W., de Jesus J.G., Ferreira N.E., Romano C.M. (2020). First case of SARS-COV-2 sequencing in cerebrospinal fluid of a patient with suspected demyelinating disease. J. Neurol..

